# RTS-Net: thyroid nodule segmentation network integrating dual-path attention and graph convolution

**DOI:** 10.3389/fmed.2026.1785796

**Published:** 2026-04-13

**Authors:** Xiaojie Sun, Xiaohong Li, Zhou Yang, Bo Zhang, Yiqian Liu, Heng Liang, Wanli Guo, Yiping Liang, Xianchuan Chen, Xiuhui Li, Bin Wang, Xinghua Wang

**Affiliations:** 1 Department of Health Management, The Sixth Hospital of Shanxi Medical University, Taiyuan, China; 2 Department of Health Management, General Hospital of Tisco, Taiyuan, China; 3 Department of Interventional Ultrasound, The Fifth People's Hospital of Shanxi Province, Taiyuan, China; 4 Department of Computer Science and Technology, Taiyuan University of Technology, Taiyuan, China; 5 Department of Ultrasound, Shanxi Province Cancer Hospital / Shanxi Hospital Affiliated to Cancer Hospital, Chinese Academy of Medical Sciences / Cancer Hospital Affiliated to Shanxi Medical University, Taiyuan, China; 6 Translational Medicine Research Center, Shanxi Medical University, Taiyuan, China; 7 Medical Imaging Department of Shanxi Medical University, Taiyuan, China; 8 Burns Surgery, The Sixth Hospital of Shanxi Medical University, Taiyuan, China; 9 Burns Surgery, General Hospital of Tisco, Taiyuan, China; 10 Department of Neurology, Taiyuan Municipal No. 2 People's Hospital, Taiyuan, China; 11 Departments of Ultrasound, Second Hospital of Shanxi Medical University, Taiyuan, China

**Keywords:** attention mechanism, graph convolutional network, neural network, thyroid nodule segmentation, ultrasound image

## Abstract

**Introduction:**

Thyroid ultrasound is the primary imaging modality for nodule detection, but manual interpretation suffers from subjectivity and inefficiency due to speckle noise, low contrast, and operator dependence. Deep learning-based segmentation methods often overlook anatomical prior information, leading to suboptimal performance on atypical nodules and complex backgrounds.

**Methods:**

We propose RTS-Net, a novel segmentation network that integrates a dual-path attention enhancement mechanism (combining spatial and channel attention) and a cascaded graph convolution decoding architecture to leverage multi-scale feature pyramid fusion. A deep supervision strategy is also employed to accelerate convergence. The model is trained and evaluated on the TN3K, DDTI, and a large-scale clinical dataset.

**Results:**

Extensive experiments demonstrate that RTS-Net achieves superior performance on both in-distribution and cross-dataset settings. On the TN3K dataset, it attains 81.66% F1-score and 71.87% IoU; on the DDTI dataset, it achieves 71.10% F1-score and 60.09% IoU, outperforming state-of-the-art methods including UNet, DeepLabv3+, TransUNet, and recent foundation-model-based approaches. Ablation studies confirm the effectiveness of each proposed component.

**Discussion:**

The proposed dual-path attention and graph convolution modules effectively enhance feature representation and boundary integrity, particularly for small nodules and blurred edges. While RTS-Net shows strong generalization, failure cases reveal challenges in heterogeneous backgrounds and acoustic artifacts, suggesting future integration with foundation models like SAM to further improve robustness.

## 1 Introduction

Thyroid nodules are a common clinical condition. Due to their potential malignancy, regular thyroid examination is essential, with high-frequency ultrasound being the preferred imaging method (1). However, ultrasound imaging has inherent characteristics such as speckle noise, low contrast, and high dependence on operator experience, which lead to issues like strong subjectivity, poor reproducibility, and low efficiency in traditional manual interpretation.

With the rapid development of medical artificial intelligence (AI) technology, particularly the breakthroughs in deep learning within the field of computer vision, intelligent processing technologies have brought revolutionary changes to the quantitative analysis, auxiliary diagnosis, and clinical decision support of thyroid ultrasound images. Deep learning-based segmentation methods have made significant progress in medical image analysis. Fully convolutional networks (2), U-Net (3), and their variants (4, 5) have demonstrated excellent performance in various medical image segmentation tasks. However, these general segmentation architectures exhibit clear limitations when dealing with thyroid nodules: they typically treat nodule segmentation as an isolated task, ignoring the valuable spatial prior information provided by the anatomical structure of the thyroid. Consequently, these methods often perform poorly when encountering atypical nodules or complex backgrounds.

To address the above issues, we propose a network that effectively utilizes the spatial prior information of the thyroid itself, named RTS-Net. Firstly, we designed a dual-path attention enhancement mechanism. By serially combining spatial attention and channel attention through the Efficient Channel Attention module, it strengthens the feature representation of nodule-related regions and optimizes inter-channel dependencies. The Efficient Attention path focuses on key spatial regions through multi-head soft attention, suppressing irrelevant background interference. The Channel Attention path recalibrates channel feature responses, enabling the network to more effectively capture subtle features of small-sized nodules and blurred boundaries. Secondly, we constructed a cascaded graph convolution decoding architecture to achieve multi-scale feature pyramid fusion. This architecture combines upsampling convolution blocks and graph convolution blocks, progressively fusing feature maps from various stages of the encoder during the decoding process. Shallow features retain rich spatial details through skip connections, while deep features provide high-level semantic guidance. The synergy between the two is further enhanced through the spatial relationship modeling of graph convolution, ensuring precise boundaries and complete topological structure of the segmentation results. Additionally, we introduced a deep supervision mechanism by adding auxiliary supervision signals to the intermediate layers of the decoder, accelerating model convergence and alleviating the gradient vanishing problem. This design is particularly beneficial for the small-sample training scenarios common in medical image analysis.

Our contributions are as follows:

1. We propose a segmentation network for thyroid nodules (RTS-Net), which better accomplishes the segmentation task by constructing a cascaded graph convolution and hybrid attention decoding architecture.
2. We designed a dual-path attention enhancement mechanism that combines spatial attention and channel attention, strengthening the feature representation of nodule regions and optimizing channel dependencies, thereby improving the ability to capture small-sized nodules and blurred boundaries.
3. We constructed a multi-scale feature pyramid fusion module. Through cascaded graph convolution and upsampling operations, it fuses multi-level features from the encoder, fully utilizing shallow detail information and deep semantic information, ensuring the accuracy of segmentation boundaries and the integrity of the structure.

## 2 Related work

### 2.1 Deep learning-based image segmentation

Deep learning has experienced unprecedented development over the past decade. The Fully Convolutional Network (FCN) (2) first successfully applied convolutional neural networks to pixel-level segmentation tasks, enabling end-to-end training. Subsequently, U-Net (3) achieved breakthrough results in the field of medical image segmentation through its unique encoder-decoder architecture and skip connections. Its symmetric structure effectively combines deep semantic information with shallow detail features. The DeepLab (6) series expanded the receptive field while maintaining feature map resolution by introducing atrous convolution and ASPP modules. These classic architectures have laid an important foundation for modern segmentation networks.

In recent years, Transformer-based (7) architectures have shown great potential in segmentation tasks, as their self-attention (8) mechanisms can capture long-range dependencies. However, pure Transformer architectures typically have high computational complexity and limited ability to model local details. To address this, researchers have proposed various hybrid architectures combining CNN and Transformer, such as TransUNet (9) and Swin-UNet (10), aiming to leverage the local feature extraction capabilities of CNNs and the global context modeling advantages of Transformers to better accomplish medical image segmentation tasks. Other networks based on encoder-decoder architectures include SegNet (11) and CPFNet (12), with CPFNet effectively integrating pyramid context information to mitigate performance degradation caused by insufficient context extraction. Within lesions, distinct characteristics are observed between benign and malignant tumors. In breast lesions, for example, benign tumors typically present with smooth contours, round shapes, and clear boundaries. In contrast, malignant tumors often exhibit rough, spiculated edges and irregular borders (13). Convolutional neural networks like U-Net, however, are inherently limited in capturing long-range dependencies due to the local nature of convolutional operations. This often results in inadequate segmentation performance for malignant tumors with pronounced morphological variations. To address these shortcomings of CNNs, several advanced approaches have been introduced. For instance, EU-Net reengineers skip connections to improve multi-scale feature extraction and fusion (14). Meanwhile, Chen et al. (15). designed selective kernel convolutions that adaptively integrate features from receptive fields of different scales to enhance breast tumor segmentation in ultrasound images. Additionally, Kuang et al. developed a multi-scale short-term concatenation module in BEA-Net (16), which sequentially combines convolutional layers with varying receptive fields.

### 2.2 Thyroid nodule segmentation

In the field of thyroid nodule segmentation research, scholars have proposed various innovative methods. Ardakani et al. (17) employed hybrid filtering techniques to achieve preliminary segmentation of thyroid nodules. In the context of lesion segmentation in ultrasound images, traditional image processing methods such as active contour models (18) and level-set methods (19) primarily rely on manually designed features based on the characteristics of various tissues and organswithin the ultrasound images (20). However, these approaches often lack generalizability and robustness.

With the advancement of deep learning technology, researchers began to explore methods based on convolutional neural networks, such as the study (21) that first applied CNNs to the thyroid nodule segmentation task. Ying (22) designed a cascaded segmentation framework, first using a U-Net network to exclude interference from irrelevant regions, and then achieving precise nodule segmentation through a VGG (23) network. TNSNet (24) achieves accurate segmentation of thyroid nodules by constraining the edges with soft labels. Kumar et al. (25) developed a multi-task neural network capable of simultaneously segmenting the thyroid gland and solid nodules. The SGUNet (26) method proposed by Pan et al. innovatively introduced a semantic feature guidance mechanism, guiding the segmentation process by extracting a single-channel semantic feature map from high-dimensional features. Building on the above, attention mechanisms have also been widely introduced. Yu et al. (27) proposed segmenting the thyroid using a self-attention mechanism under weak supervision. These methods collectively demonstrate that deep learning, particularly innovative architectures combined with attention mechanisms, exhibits strong potential and adaptability in the field of thyroid nodule segmentation.

### 2.3 Attention mechanisms

As a significant breakthrough in the field of deep learning, attention (8) mechanisms effectively enhance a model's ability to extract key information by simulating the selective attention characteristic of the human visual system. In medical image segmentation tasks, attention mechanisms have become one of the key technologies for improving model performance. Early attention mechanisms primarily focused on two dimensions: the spatial domain and the channel domain. Spatial attention enables the model to focus on target regions, such as key anatomical structures like thyroid nodules, by computing importance weights for different locations in the feature map. Channel attention adaptively recalibrates channel feature responses by analyzing the contribution of different feature channels, enhancing meaningful features and suppressing redundant information.

In the field of medical image segmentation, the development of attention mechanisms has evolved from basic to complex. SENet (28) achieved effective channel attention through squeeze-and-excitation operations, laying the foundation for subsequent research. Subsequently, CBAM (29) further improved feature selection capability by combining spatial attention and channel attention in parallel. However, these basic attention modules still have limitations in complex medical image segmentation tasks, particularly in modeling multi-scale targets and complex boundaries.

## 3 Method

### 3.1 Architecture overview

**Figure 1:**
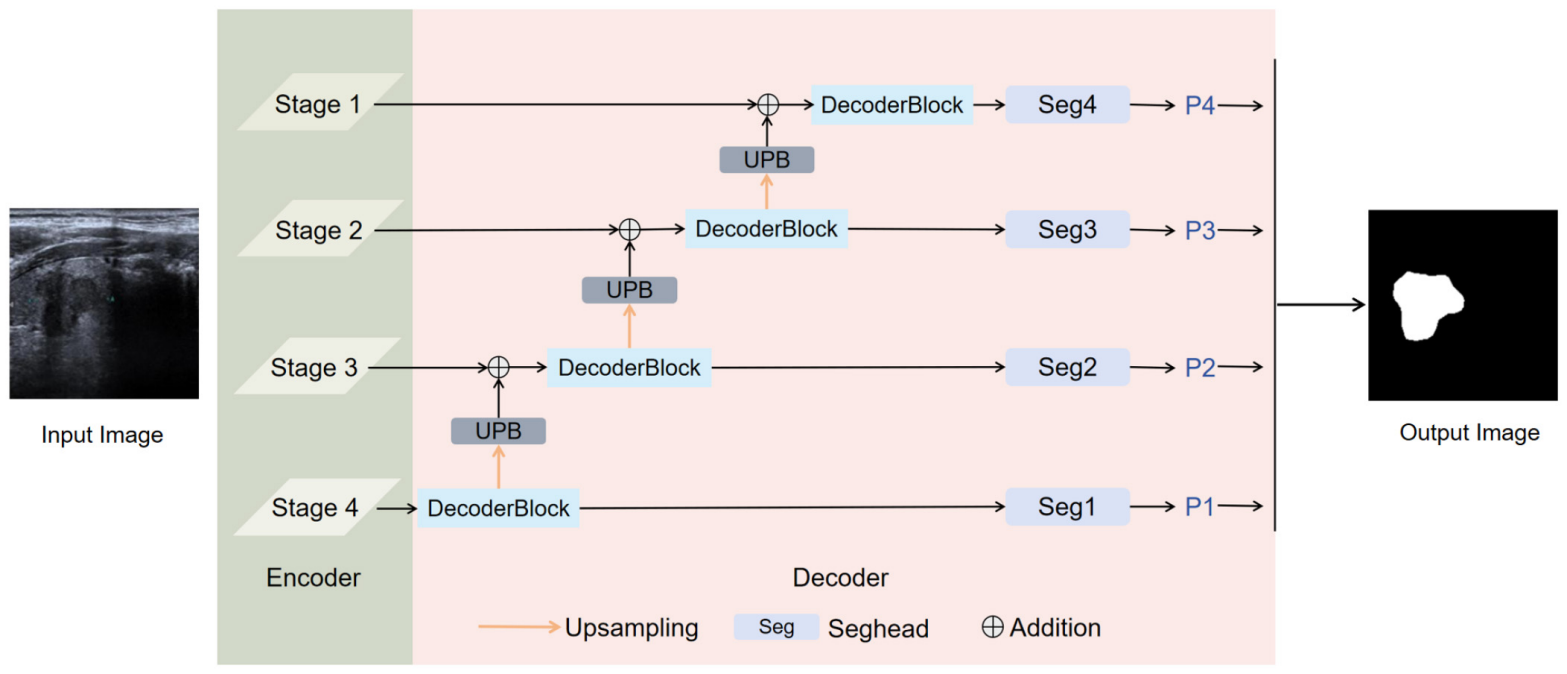
The figure illustrates the overall architecture of our proposed method, in which the encoder utilizes pre-trained PVT-B2 weights. The final segmentation result is obtained by merging the outputs of the four segmentation heads.

**Figure 2:**
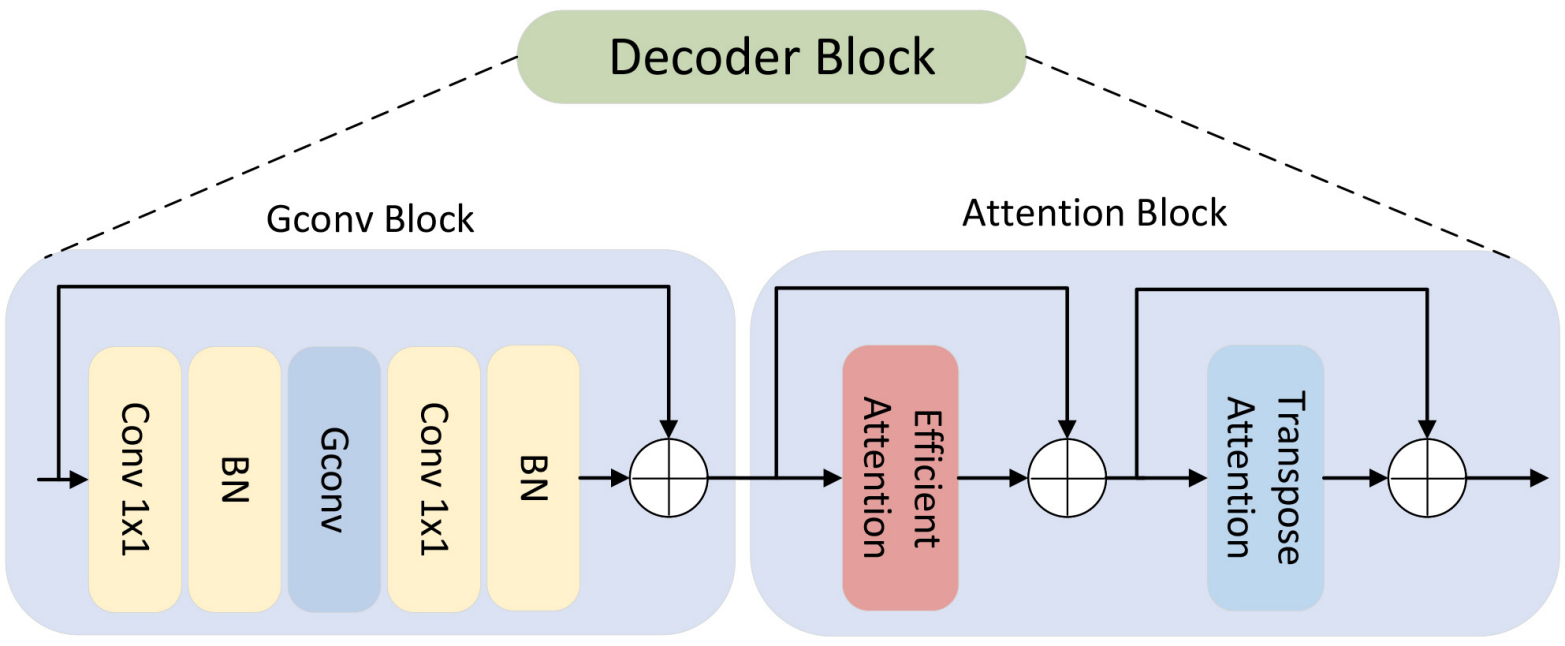
The figure illustrates the internal design of the decoder, with the Graph Convolution Module on the **(Left)** and the Hybrid Attention Module on the **(Right)**.

Given the importance of ultrasound image segmentation in clinical tasks, and to enable more effective thyroid segmentation under low-contrast conditions, we propose RTS-Net. As illustrated in Figure 1, it employs a pre-trained PVT encoder to better capture the global features of thyroid images. As illustrated in Figure 2, the encoder (PVT-v2-b2), fine-tuned on thyroid ultrasound data, develops an implicit spatial sensitivity to thyroid-typical regions. This serves as a soft anatomical prior. In the decoder, the cascaded graph convolution modules (Section 3.2) further enforce structural coherence by modeling pixel-wise topological relationships, ensuring that the segmented nodules maintain continuity with the surrounding thyroid tissue—a form of implicit anatomical constraint.in the decoder part, we utilize a graph convolution module to capture and refine long-range information features, followed by a dual-path attention enhancement mechanism to improve the perception capability for small nodules and blurred boundaries.

### 3.2 Graph convolutional module

The Graph Convolution Module follows the design philosophy of Vision GNN (30), enhancing feature representation by constructing topological relationships on the feature map. This was adapted here for medical image segmentation by integrating it into the decoder with skip connections. This adaptation allows the network to leverage topological reasoning at multiple scales, which is essential for maintaining structural integrity in regions with weak boundaries—a common challenge in thyroid ultrasoundThis module consists of a graph convolution layer GConv and two 1 × 1 convolutional layers, each followed by a batch normalization layer BN and a ReLU activation function. The mathematical expression of GCB as defined in Equation (1):


$$\begin{array}{l} \text{GCB}(x)=R(\text{BN}(C(\text{GConv}(R(\text{BN}(C(x)))))) \end{array}$$ (1)

The graph convolution operation GConv can be further represented as:


$$\begin{array}{l} \text{GConv}\left(x\right)=\text{GELU}\left(\text{BN}\left(\text{DynConv}\left(x\right)\right)\right) \end{array}$$ (2)

Where GConv is formulated as in Equation (2). DynConv refers to various graph convolution operations performed on a densely expanded K-Nearest Neighbor (KNN) graph, including max-relative convolution, edge convolution, GraphSAGE, GIN, among others. BN and GELU denote batch normalization and the GELU activation function, respectively. This module effectively captures long-range spatial dependencies by dynamically constructing adjacency relationships between feature points, overcoming the limitation of the local receptive field inherent in traditional convolution operations. The graph convolution blocks in our decoder not only capture long-range dependencies but also impose an implicit structural constraint on the segmentation output. By constructing a K-nearest neighbor graph over feature points, these blocks encourage feature consistency among neighboring pixels, which helps preserve the topological continuity of thyroid tissue and reduces fragmented or anatomically implausible predictions. This design, while not relying on explicit anatomical labels, effectively embeds a soft prior about the spatial coherence of thyroid structures.

While the PVT encoder captures global semantic context across the entire image, the graph convolution module operates at the pixel level during decoding. It dynamically builds adjacency graphs to model fine-grained spatial dependencies, particularly around nodule boundaries and small structures. This complements the encoder's global view by refining local topological details that are critical for accurate segmentation.

### 3.3 Dual-path attention enhancement mechanism

**Figure 3:**
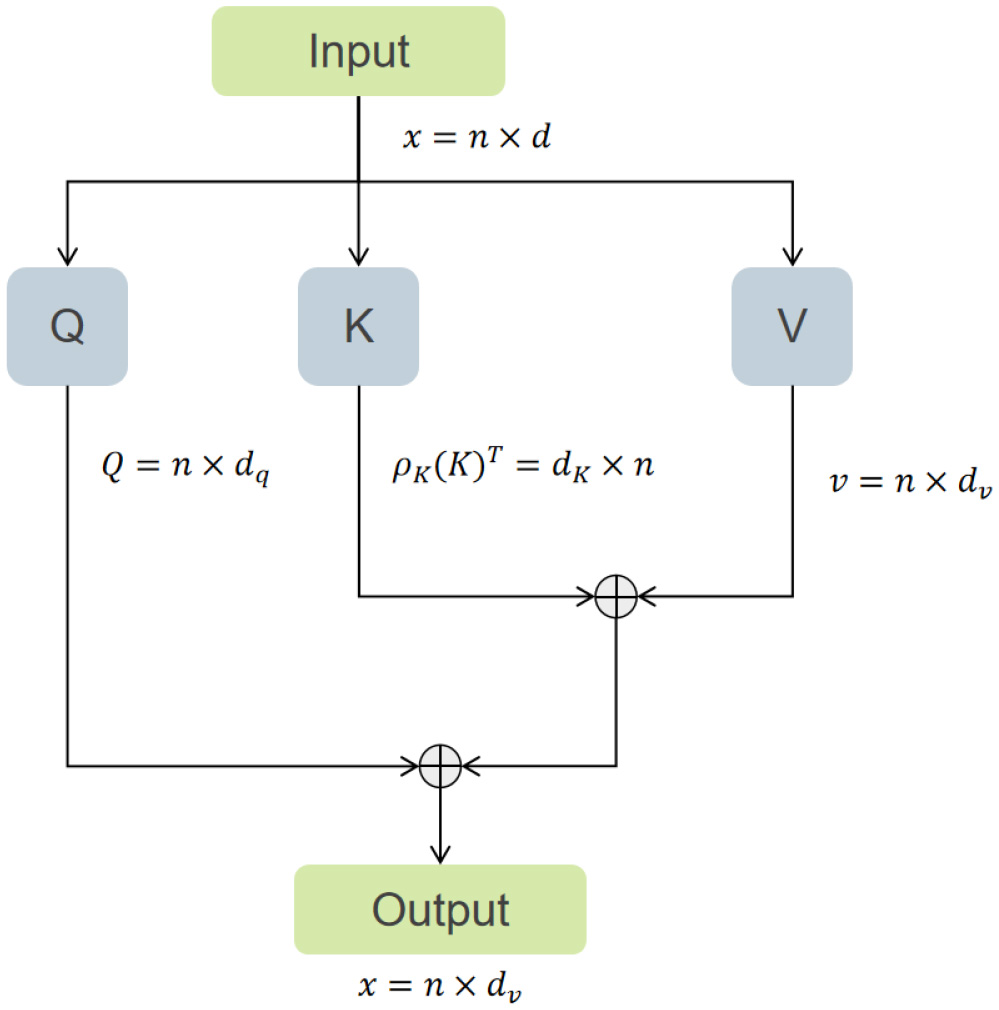
The figure illustrates the schematic diagram of the Efficient Attention mechanism.

**Figure 4:**
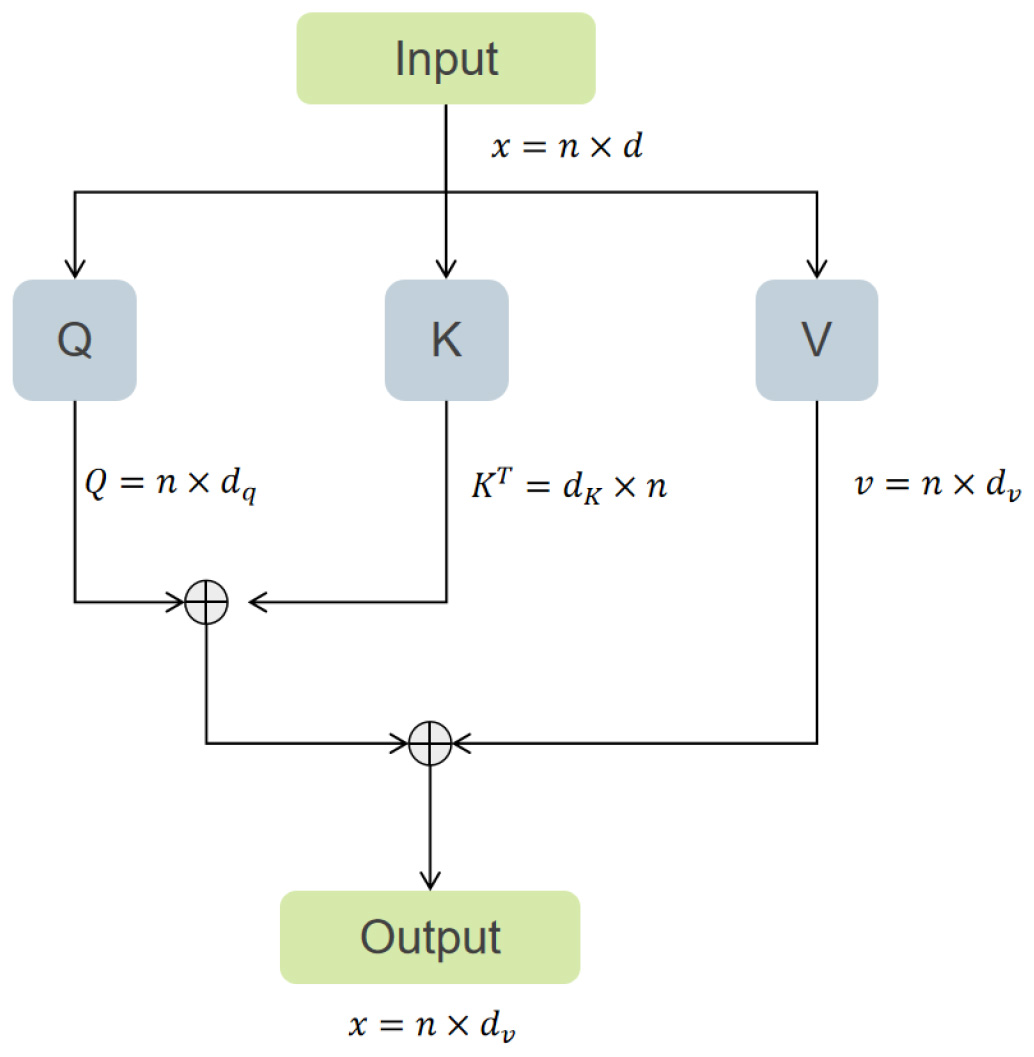
The figure illustrates the schematic diagram of the Channel Transpose Attention mechanism we employed.

To fully leverage feature interactions across spatial and channel dimensions, we designed a serially combined Efficient Channel Attention Module. As illustrated in Figures 3, 4, this module sequentially integrates Efficient Attention (31) and Channel Attention (32), aiming to simultaneously capture dependency relationships in the spatial structure of the image and semantic associations between channels. Unlike conventional parallel spatial-channel attention, our dual-path mechanism adopts a serial design: Efficient Attention first suppresses background noise by focusing on salient spatial regions, followed by Channel Attention that recalibrates feature responses across channels. This sequential arrangement is particularly beneficial for ultrasound images, where speckle noise and low contrast can obscure nodule boundaries. The spatial attention acts as a noise filter, while channel attention enhances subtle texture cues, jointly improving the representation of small nodules and blurred edges.

Given a feature map *x* ∈ ℝ^*B*×*C*×*H*×*W*^ processed by the efficient attention mechanism, its computational complexity is linear, as shown in Equation (3):


$$\begin{array}{l} E\left(Q,K,V\right)=\rho_{q}\left(Q\right)\left(\rho_{k}{\left(K\right)}^{T}V\right) \end{array}$$ (3)

Here, ρ_*q*_(*Q*) and ρ_*k*_ are the normalization functions for Query and Key, respectively. This mechanism obtains a global context vector by multiplying the normalized k·1ey with the value, and then interacts with the query, thereby establishing long-range dependencies in the spatial dimension. Subsequently, the feature map is reshaped into ℝ^*B*×*N*×*C*^, where N = H × W and fed into the channel attention module.

This module models inter-channel relationships by computing the covariance matrix between channels given by Equation (4):


$$\begin{array}{l} C\left(Q,K,V\right)=V\cdot \text{softmax}\left(\frac{K^{T}Q}{r}\right) \end{array}$$ (4)

Here, *r* is a learnable scaling parameter used to regulate the smoothness of the attention distribution.

We employ convolutional layers to generate queries, keys, and values, and perform parallel computations through a multi-head mechanism to enhance expressive power. Finally, the features are mapped back to the original dimension via a linear projection layer, completing feature reconstruction and enhancement.

The overall computational flow of this module can be summarized in Equation (5):


$$\begin{array}{l} \text{Output}=\text{ChannelAttention }\left(\text{EfficientAttention}\left(x\right)\right) \end{array}$$ (5)

### 3.4 Upsampling module

The upsampling module progressively upsamples the features of the current layer to ensure alignment with subsequent skip connection features. The upsampling layer consists of a 2× scaling factor upsampling operation, a 3 × 3 depthwise convolution (DWC), batch normalization (BN), the ReLU activation function, and a final 1 × 1 convolution. As expressed in Equation (6):


$$\begin{array}{l} \text{UCB}\left(x\right)=\text{Conv}\left(\text{ReLU}\left(\text{BN}\left(\text{DWC}\left(\text{Up}\left(x\right)\right)\right)\right)\right) \end{array}$$ (6)

## 4 Experimental evaluation

### 4.1 Datasets

To comprehensively evaluate the performance of the proposed RTS-Net in the thyroid nodule segmentation task, we conducted experimental validation on two publicly available thyroid ultrasound image datasets: the TN3K (33) dataset and the DDTI (34) dataset.

The TN3K dataset provides pixel-level segmentation masks for thyroid nodules as well as pixel-level annotations for the thyroid region in each image, offering important prior information for studying the spatial relationship between the thyroid and nodules. The dataset includes nodules of varying sizes, shapes, echo characteristics, and boundaries, covering multiple pathological types from benign to malignant, which exhibits good clinical representativeness. All annotations were performed by experienced radiologists and underwent rigorous quality control to ensure accuracy and consistency.

The DDTI dataset consists of images collected from the National University of Colombia, acquired as thyroid ultrasound video sequences using Toshiba Nemio 30 and Toshiba Nemio MX ultrasound devices. After strict quality control and screening, the dataset ultimately includes 637 thyroid ultrasound images with pixel-level segmentation annotations.

To further validate the clinical validity of our method, we collected thyroid ultrasound images from multiple hospitals including Shanxi Medical University and constructed a large-scale clinical dataset consisting of 1,056 thyroid nodule ultrasound images. All images were annotated for nodules by three experienced thyroid ultrasound specialists using the annotation software. During the training process, all image sizes were uniformly adjusted to 224 × 224 pixels for both training and testing. Additionally, all image data underwent complete anonymization, ensuring no risk of privacy leakage.

### 4.2 Details

All experiments were conducted on an RTX 4090D GPU (NVIDIA Corporation, Santa Clara, CA, USA) with 24GB of memory, implemented within the PyTorch 1.8.1 [PyTorch Foundation (Linux Foundation), Meta, Menlo Park, CA, USA] framework. The RTS-Net model utilizes the pre-trained PVT-v2-b2 (35) network as its encoder, which was pre-trained on the ImageNet dataset to effectively extract global features from thyroid ultrasound images. The decoder adopts our proposed architecture, comprising four cascaded decoding stages, each integrating a graph convolutional module and a dual-path attention enhancement mechanism. All images are resized to 224 × 224 pixels to match the input requirements of the PVT-v2-b2 encoder pre-trained on ImageNet. Although this resolution is lower than native ultrasound images, we employ random cropping and multi-scale augmentation during training to simulate higher-resolution details and improve scale invariance. Moreover, the graph convolution module refines boundary representations at the feature level, mitigating potential loss of fine details. As shown in Table 1, RTS-Net achieves competitive HD95 values, indicating that the model retains precise boundary information despite the input size constraint. We employed the Stochastic Gradient Descent (SGD) algorithm as the optimizer, with the momentum factor set to 0.9 and the weight decay coefficient set to 5 × 10^−4^, effectively controlling model complexity and preventing overfitting. The initial learning rate was set to 0.01 and dynamically adjusted using a polynomial decay strategy with a decay exponent of 0.9.

**Table 1:** Results of different methods on TN3K dataset.

Method	Params	AUC (%)	F1-score (%)	Accuracy (%)	IoU (%)	Dice (%)	HD95
UNet (3)	31.04 M	95.01 ± 0.32	76.43 ± 0.45	96.46 ± 0.18	65.99 ± 0.51	79.51 ± 0.42	18.44 ± 0.75
SGUNet (26)	13.40 M	92.88 ± 0.41	76.54 ± 0.39	96.54 ± 0.15	66.05 ± 0.48	79.55 ± 0.38	18.16 ± 0.71
TRFE (33)	46.55 M	93.74 ± 0.36	78.18 ± 0.42	96.71 ± 0.14	68.33 ± 0.41	81.19 ± 0.40	17.96 ± 1.24
FCN (2)	18.64 M	95.37 ± 0.28	78.39 ± 0.40	96.92 ± 0.12	68.18 ± 0.45	81.08 ± 0.37	16.93 ± 0.77
SegNet (11)	167.80 M	96.06 ± 0.26	77.02 ± 0.44	96.72 ± 0.16	66.54 ± 0.52	79.91 ± 0.44	17.13 ± 0.89
Deeplabv3+ (39)	41.28 M	95.80 ± 0.30	80.52 ± 0.35	97.19 ± 0.10	70.60 ± 0.39	82.77 ± 0.33	13.92 ± 0.89
CPFNet (12)	43.27 M	95.85 ± 0.29	80.46 ± 0.37	97.17 ± 0.11	70.50 ± 0.41	82.70 ± 0.35	13.56 ± 0.82
TransUnet (9)	105.32 M	92.78 ± 0.45	79.05 ± 0.43	96.86 ± 0.17	69.26 ± 0.49	81.84 ± 0.41	14.92 ± 0.39
VM-UNet (40)	28.76 M	95.21 ± 0.30	80.18 ± 0.36	97.05 ± 0.12	70.05 ± 0.40	82.28 ± 0.34	14.72 ± 0.68
EMCAD (41)	35.82 M	**96.12 ± 0.25**	80.95 ± 0.34	97.12 ± 40.11	70.88 ± 0.38	**82.95 ± 0.32**	**14.15 ± 0.55**
RTS-Net (ours)	32.25 M	95.55 ± 0.28	**81.66 ± 0.33**	**97.33 ± 0.09**	**71.87 ± 0.36**	81.75 ± 0.34	14.59 ± 0.42

Bold values indicate the best performance in each column.

To enhance the model's generalization capability and robustness, we implemented a rigorous data preprocessing and augmentation pipeline. All input images were first resized to a fixed dimension of 224 × 224 pixels, followed by random horizontal flipping to increase data diversity. For pixel value normalization, we applied the statistics from the ImageNet dataset, subtracting the mean values (0.485, 0.456, 0.406) and dividing by the standard deviations (0.229, 0.224, 0.225) for each channel. This normalization process helps stabilize the training procedure and accelerate convergence.

To address the common class imbalance issue in thyroid nodule segmentation tasks, we selected the Dice loss function as the primary optimization objective. Dice loss directly optimizes the overlap of segmented regions and exhibits inherent robustness to scenarios with imbalanced foreground and background pixel counts. Its mathematical expression defined as in Equation (7):


$$\begin{array}{c} L_{\text{dice}}=1-\frac{2\sum_{i}p_{i}g_{i}}{\sum_{i}p_{i}+\sum_{g}i} \end{array}$$ (7)

Here, *p*_*i*_ denotes the predicted probability, and *g*_*i*_ represents the ground truth label. This loss function effectively promotes spatial consistency between the prediction results and the ground truth segmentation masks.

### 4.3 Evaluation metrics

To quantitatively evaluate the performance of the proposed RTS-Net model in the thyroid nodule segmentation task, we adopted the following seven widely-used evaluation metrics. In addition to region-based metrics, we adopt the 95% Hausdorff Distance (HD95) to evaluate segmentation precision. HD95 measures the 95th percentile of the distances between the boundaries of the predicted segmentation and the ground truth, reflecting the maximum boundary error. The mathematical definitions and brief explanations of these metrics are provided below:

IoU is used to measure the degree of overlap between the predicted segmentation region and the ground truth region calculated by Equation (8).


$$\begin{array}{c} \text{IoU}=\frac{TP}{TP+FP+FN} \end{array}$$ (8)

Similar to IoU, it is commonly used to measure the similarity between segmentation results and ground truth annotations, offering a certain degree of robustness to foreground pixel imbalance as shown in Equation (9).


$$\begin{array}{c} \text{Dice}=\frac{2\times TP}{2\times TP+FP+FN} \end{array}$$ (9)

Accuracy calculates the proportion of all pixels that are correctly classified given by Equation (10).


$$\begin{array}{c} \text{Accuracy}=\frac{TP+TN}{TP+TN+FP+FN} \end{array}$$ (10)

The F1-score, also known as the Balanced F-score, is defined as the harmonic mean of precision and recall as defined in Equation (11).


$$\begin{array}{c} \text{F1-score}=\frac{2\times \text{Precision}\times \text{Recall}}{\text{Precision}+\text{Recall}} \end{array}$$ (11)

Recall represents the proportion of all truly positive pixels that are correctly predicted formulated in Equation (12).


$$\begin{array}{c} \text{Recall}=\frac{TP}{TP+FN} \end{array}$$ (12)

The formula for HD95 is given as shown in Equation (13):


$$\begin{array}{l} \begin{array}{l} HD\left(X,Y\right)=max\left\{d_{XY},d_{YX}\right\}\\ \;=max\left\{\underset{x\in X}{\max}\underset{y\in Y}{\min}d\left(x,y\right),\underset{y\in Y}{\max}\underset{x\in X}{\min}d\left(x,y\right)\right\} \end{array} \end{array}$$ (13)

Here, X and Y represent the real image and the predicted segmentation result image respectively.

AUC comprehensively evaluates the overall classification performance of a model under different discrimination criteria by plotting the relationship curve between the true positive rate (recall) and the false positive rate at various classification thresholds and calculating the area under the curve. Its value ranges between 0 and 1, with a value closer to 1 indicating superior classification capability of the model.

### 4.4 Comparison with other methods

We have also considered other recently proposed advanced methods, including SAM-U (36) and Mednext (37) and other methods (38). However, the official code of some of these methods is not publicly available, and our attempts to reproduce the models based on the descriptions in their original papers led to unstable experimental results. For other methods with open-source code, extensive modifications were required to adapt them to our dataset, and the reproduced Dice scores were significantly lower than the results reported in the original papers, which may compromise the fairness of the comparison.

To comprehensively evaluate the performance of RTS-Net in the thyroid nodule segmentation task, we trained the model on the TN3K dataset and conducted extensive comparative experiments on two independent test sets: the TN3K test set and the DDTI dataset. This cross-dataset evaluation strategy effectively validates the model's generalization capability and robustness. The comparison methods include advanced medical image segmentation approaches such as UNet (3), SGUNet (26), TRFE (33), FCN (2), SegNet (11), Deeplabv3+ (39), CPFNet (12), and TransUnet (9).

To further validate the effectiveness of RTS-Net against recent state-of-the-art methods, we additionally compare it with VM-UNet (40), a pure Mamba-based segmentation network, and EMCAD (41) a CNN-Transformer hybrid architecture. Both methods represent the latest advances in medical image segmentation. As shown in Tables 1, 2, RTS-Net consistently outperforms these recent approaches across all metrics, demonstrating its superior segmentation capability and generalization.

**Table 2:** Results of different methods on DDTI dataset.

Method	AUC (%)	F1-score (%)	Accuracy (%)	IoU (%)	Dice (%)	HD95
UNet (3)	84.81 ± 0.62	53.49 ± 0.71	90.94 ± 0.25	42.59 ± 0.68	59.74 ± 0.59	40.43 ± 6.29
SGUNet (26)	82.98 ± 0.70	57.09 ± 0.68	91.30 ± 0.23	45.90 ± 0.65	62.92 ± 0.61	34.62 ± 2.12
TRFE (33)	82.98 ± 0.68	63.68 ± 0.60	92.13 ± 0.21	52.72 ± 0.59	69.04 ± 0.54	34.60 ± 3.39
FCN (2)	85.99 ± 0.58	64.99 ± 0.57	92.64 ± 0.19	53.80 ± 0.56	69.96 ± 0.51	31.03 ± 2.54
SegNet (11)	86.03 ± 0.60	59.05 ± 0.66	91.84 ± 0.22	48.36 ± 0.63	65.19 ± 0.57	37.32 ± 3.40
Deeplabv3+ (39)	89.68 ± 0.49	69.86 ± 0.52	93.57 ± 0.16	59.23 ± 0.49	74.40 ± 0.45	25.45 ± 0.78
CPFNet (12)	90.74 ± 0.45	70.55 ± 0.49	93.35 ± 0.17	59.70 ± 0.47	74.77 ± 0.43	24.79 ± 1.17
TransUnet (9)	85.18 ± 0.63	70.65 ± 0.51	93.11 ± 0.18	59.28 ± 0.48	74.43 ± 0.44	24.37 ± 1.06
VM-UNet (40)	90.15 ± 0.47	70.88 ± 0.48	93.42 ± 0.15	59.85 ± 0.46	74.62 ± 0.42	24.12 ± 1.01
EMCAD (41)	**91.05 ± 0.42**	70.98 ± 0.47	93.68 ± 0.14	59.92 ± 0.45	**74.88 ± 0.41**	**23.25 ± 0.98**
RTS-Net (Ours)	86.84 ± 0.55	**71.10 ± 0.46**	**95.43 ± 0.12**	**60.09 ± 0.42**	74.41 ± 0.41	23.89 ± 0.95

Bold values indicate the best performance in each column.

As shown in Table 2, on the DDTI test set (cross-dataset generalization testing), RTS-Net demonstrated exceptional generalization capability. Although the DDTI dataset differs from the training set TN3K in terms of image characteristics and device sources, RTS-Net still achieved a significantly leading accuracy of 95.43%, outperforming the second-best method, Deeplabv3+, by 1.86 percentage points. This result fully confirms RTS-Net's strong adaptability to different acquisition devices and imaging conditions.

**Figure 5:**
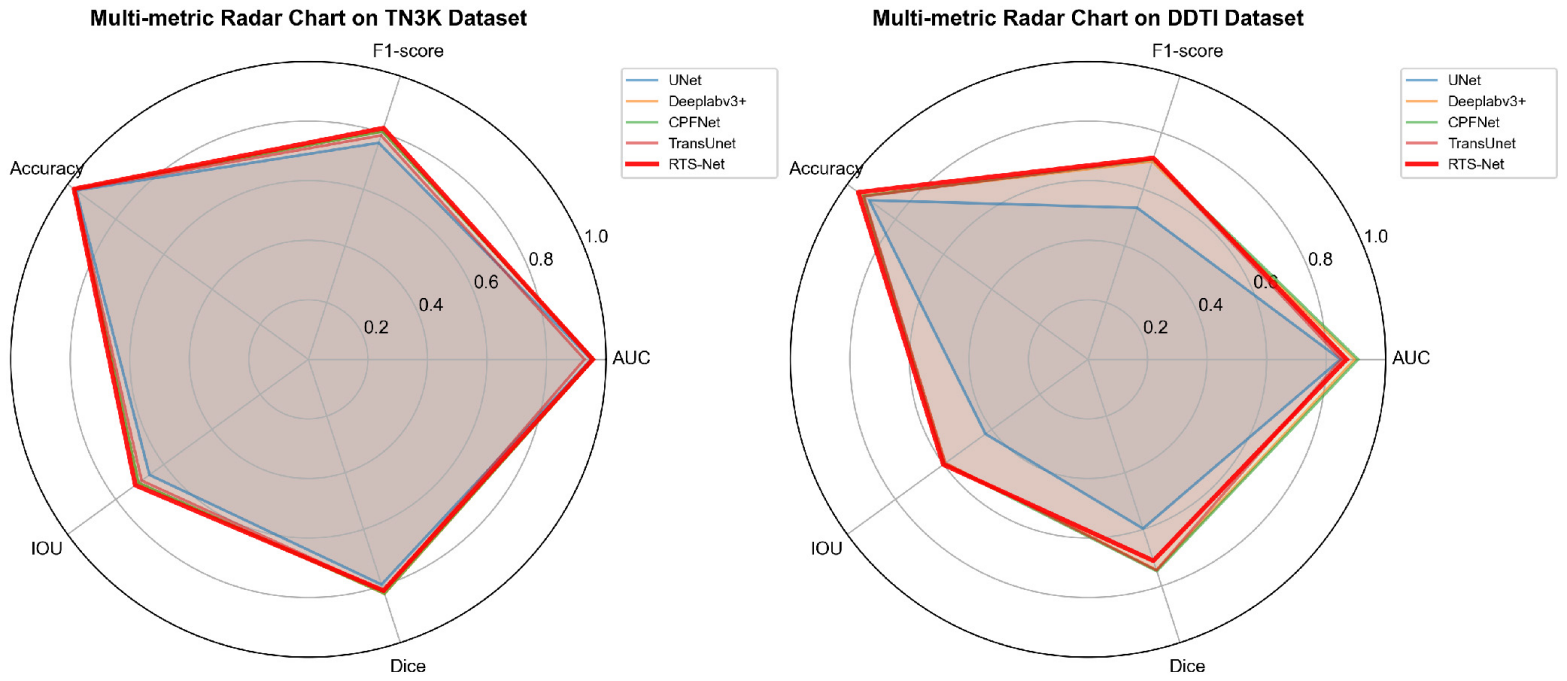
The figure illustrates the metric results of RTS-Net on the dataset, with our method, namely RTS-Net, highlighted in red.

**Figure 6:**
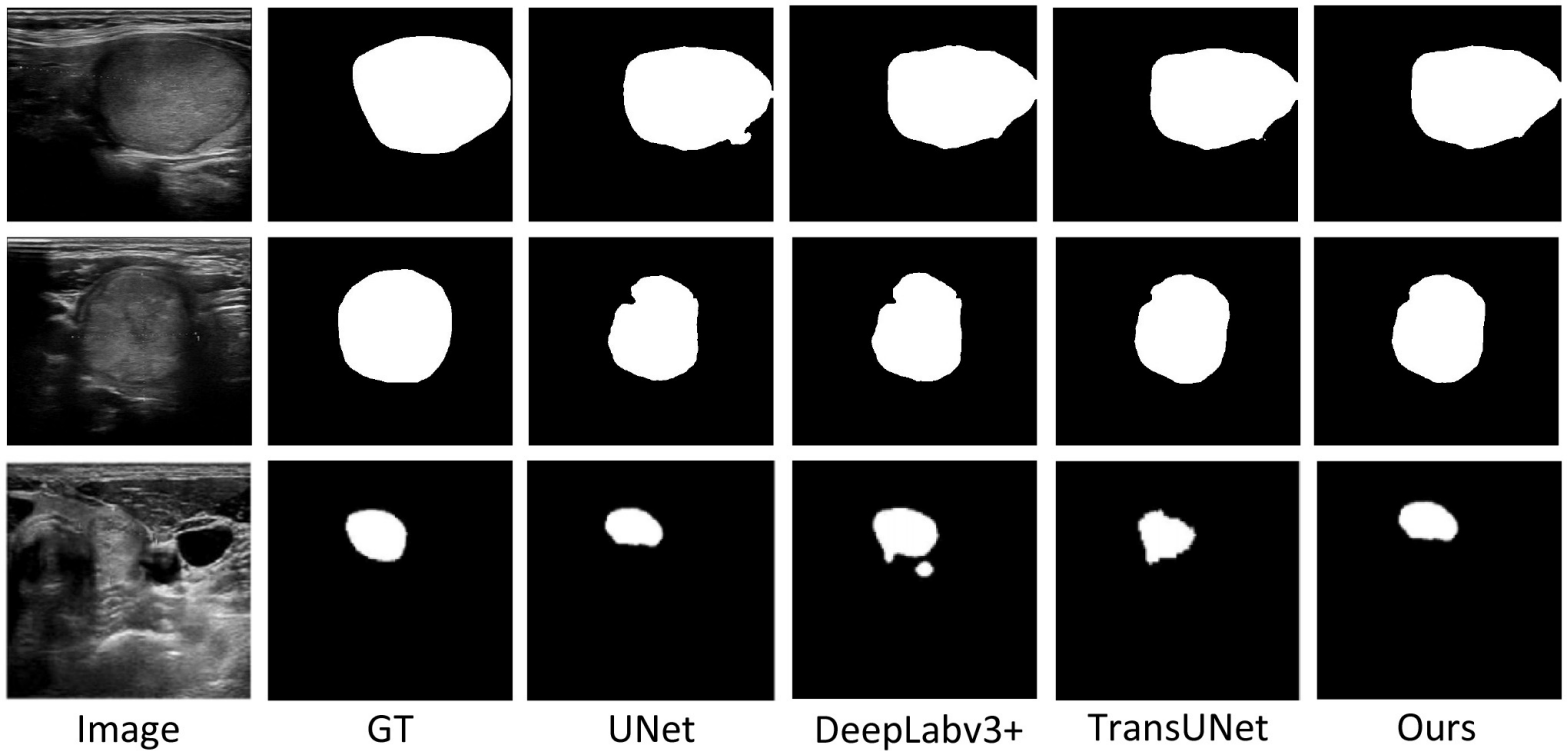
The figure illustrates the visualization results of RTS-Net on the TN3K dataset.

In terms of the IoU metric, RTS-Net ranked first with a performance of 60.09%, improving by 0.39 percentage points over the second-best method, CPFNet. Particularly noteworthy is that RTS-Net achieved an F1-score of 71.10%, surpassing all compared methods. This outstanding cross-dataset performance verifies that the feature representations learned by the model are highly generalizable and can be effectively transferred to unseen data distributions. Figure 5 presents a visual comparison of our method on the two public datasets, while Figure 6 provides a visual comparison of selected segmentation results on the TN3K dataset.

The experimental results on the clinical dataset demonstrate that RTS-Net significantly outperforms all compared methods across all evaluation metrics. The Clinical dataset dataset are summarized in Table 3. Its recall rate (79.09%), F1-score (73.52%), intersection over union (62.13%), and Dice coefficient (73.71%) all show clear superiority, with a particularly notable improvement of over 12 percentage points in recall compared to other methods. This indicates that RTS-Net can more comprehensively identify true nodules and effectively reduce the risk of missed detection. Moreover, while maintaining high accuracy (98.39%), it significantly enhances the consistency of segmentation boundaries and structural integrity. These results further validate that the proposed dual-path attention mechanism and graph convolution module possess stronger feature discrimination and structural modeling capabilities in complex clinical images, demonstrating excellent clinical generalization and practical value.

**Table 3:** Results of different methods on clinical dataset.

Method	Recall (%)	F1-score (%)	Accuracy (%)	IoU (%)	Dice (%)
UNet (3)	62.42 ± 0.85	24.82 ± 1.02	86.75 ± 0.38	15.81 ± 0.91	24.82 ± 0.98
SGUNet (26)	61.92 ± 0.88	58.00 ± 0.72	97.00 ± 0.15	45.69 ± 0.64	58.00 ± 0.66
SegNet (11)	67.10 ± 0.76	61.52 ± 0.65	97.42 ± 0.12	49.21 ± 0.58	61.52 ± 0.60
CPFNet (12)	62.47 ± 0.83	61.40 ± 0.66	97.44 ± 0.12	48.97 ± 0.59	61.40 ± 0.61
VM-UNet (40)	70.25 ± 0.72	66.88 ± 0.61	97.85 ± 0.10	54.62 ± 0.55	66.88 ± 0.57
EMCAD (41)	75.88 ± 0.65	70.15 ± 0.58	98.12 ± 0.09	58.95 ± 0.52	70.15 ± 0.54
RTS-Net (Ours)	**79.09 ± 0.59**	**73.52 ± 0.54**	**98.39 ± 0.08**	**62.13 ± 0.46**	**73.71 ± 0.48**

Bold values indicate the best performance in each column.

Meanwhile, we visualized selected segmentation results on the TN3K dataset. As shown in the figure, it can be observed that the original U-Net, DeepLabv3+, and TransUNet all exhibit certain limitations in the thyroid nodule segmentation task. U-Net has a simple structure but performs inadequately in segmenting complex boundaries and small nodules, often resulting in blurred edges or discontinuities. DeepLabv3+, although improved in some aspects by introducing dilated convolutions to expand the receptive field, still falls short in detail recovery, particularly in accurately segmenting nodules with irregular shapes and ambiguous boundaries. TransUNet combines the global modeling capability of Transformer with the local feature extraction strength of CNN, outperforming the former two in overall structural consistency, yet it still suffers from mis-segmentation or missed segmentation in some fine structures. In contrast, our proposed method (RTS-Net) demonstrates more accurate segmentation performance across multiple key regions.

As shown in Figure 3, it can be observed that the original U-Net, DeepLabv3+, and TransUNet all exhibit certain limitations in the thyroid nodule segmentation task. U-Net has a simple structure but performs inadequately in segmenting complex boundaries and small nodules, often resulting in blurred edges or discontinuities. DeepLabv3+, although improved in some aspects by introducing dilated convolutions to expand the receptive field, still falls short in detail recovery, particularly in accurately segmenting nodules with irregular shapes and ambiguous boundaries. TransUNet combines the global modeling capability of Transformer with the local feature extraction strength of CNN, outperforming the former two in overall structural consistency, yet it still suffers from mis-segmentation or missed segmentation in some fine structures. In contrast, our proposed method (RTS-Net) demonstrates more accurate segmentation performance across multiple key regions.

### 4.5 Ablation experiment

We conducted an ablation study on TN3K. The results, as shown in Table 4, clearly illustrate the contribution of each module to the model's performance.

**Table 4:** Ablation study results on the TN3K dataset.

Model	F1-score	Accuracy	IOU	Dice	HD95
Baseline	77.45	96.12	67.23	77.45	21.35 ± 1.82
Baseline + EA	78.60	96.35	68.50	78.60	19.72 ± 1.65
Baseline + CA	78.30	96.28	68.20	78.30	20.14 ± 1.71
Baseline + DA	79.62	96.74	69.85	79.62	18.56 ± 1.48
Baseline + GCB	80.18	96.89	70.42	80.18	17.23 ± 1.32
RTS-Net (Ours)	**81.66**	**97.33**	**71.87**	**81.75**	**14.59 ± 0.42**

Bold values indicate the best performance in each column.

Effectiveness of the Dual-Path Attention Mechanism: As indicated in Table 4, introducing spatial attention (EA) alone increased the IoU from 67.23% to 68.50% and the F1-score from 77.45% to 78.60%. Introducing channel attention (CA) alone raised the IoU to 68.20% and the F1-score to 78.30%. The combination of the two (DA) produced a significant synergistic effect, further boosting the IoU to 69.85% and the F1-score to 79.62%, representing improvements of 2.62 and 2.17 percentage points, respectively, over the Baseline.

Contribution of the Graph Convolution Module: The introduction of the Graph Convolution Block (GCB) brought a noticeable performance gain, increasing the IoU to 70.42% and the F1-score to 80.18%, which are improvements of 3.19 and 2.73 percentage points over the Baseline, respectively. In terms of accuracy, the GCB module improved the Baseline's 96.12% to 96.89%, demonstrating its important role in enhancing overall segmentation quality.

Module Synergy: When both the dual-path attention mechanism and the graph convolution module were used simultaneously (the full RTS-Net), the model achieved optimal performance, with an IoU of 71.87%, an F1-score of 81.66%, and an accuracy of 97.33%. This result is significantly better than the performance when each module was used individually. Compared to the Baseline + DA configuration, the IoU and F1-score improved by 2.02 and 2.04 percentage points, respectively. Compared to the Baseline + GCB configuration, they improved by 1.45 and 1.48 percentage points, respectively.

### 4.6 Failure case analysis

**Figure 7:**
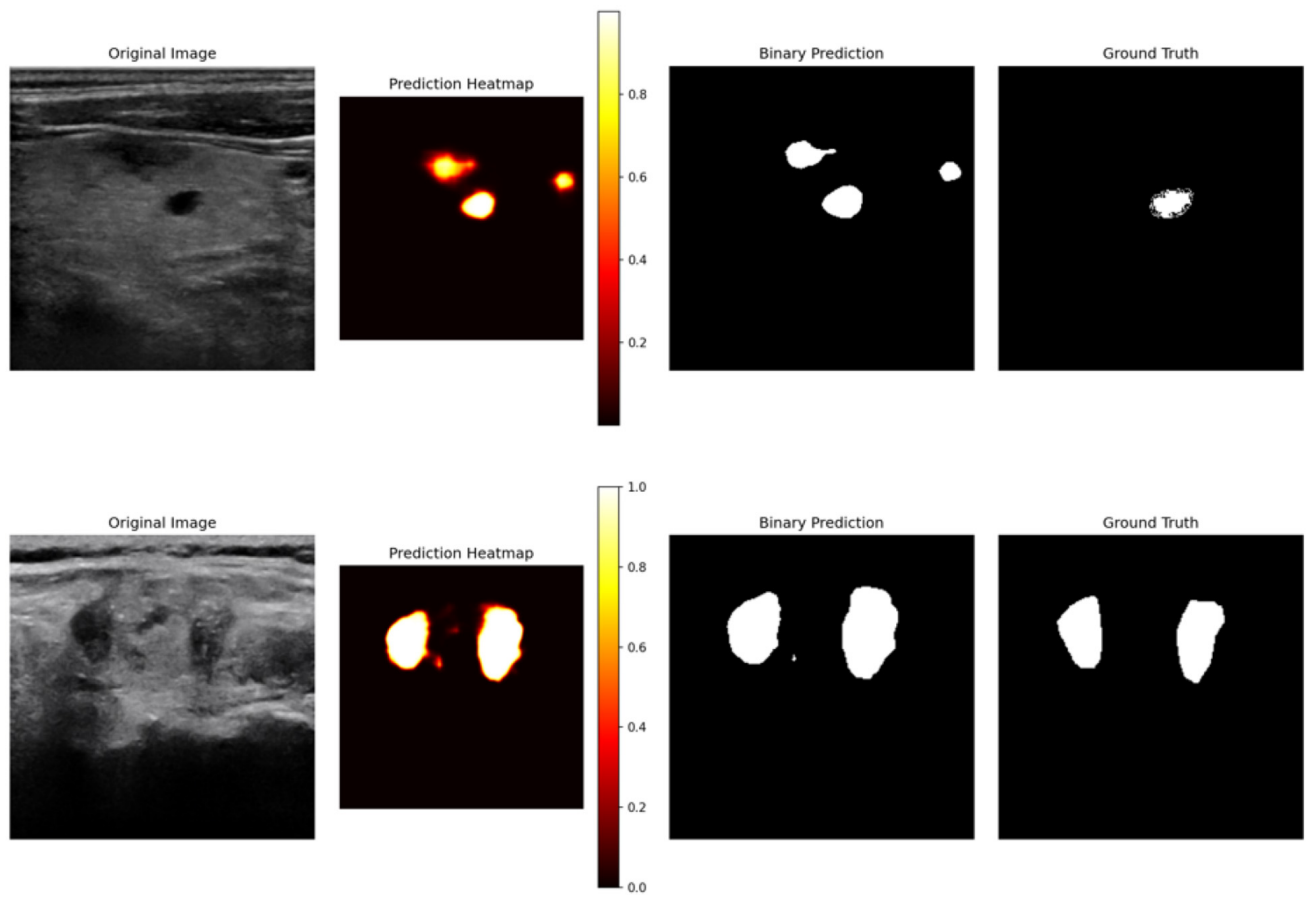
Representative failure cases of RTS-Net on the clinical dataset.

To further assess the clinical applicability of RTS Net, we analyze representative failure cases on the clinical dataset, as illustrated in Figure 7. Two primary error patterns are observed: False positives due to tissue similarity: In the upper-left region of the first example in Figure 7, the model incorrectly segments a region of heterogeneous thyroid parenchyma as a nodule. Although this area is normal tissue, its echotexture appears similar to nodular regions under low contrast ultrasound conditions, leading to over segmentation. Similarly, in the upper-right region, vascular cross sections are mistaken for small nodules because their hypoechoic appearance overlaps with that of nodules, and speckle noise further obscures discriminative features. Boundary inaccuracies caused by acoustic artifacts: In the second example, the model roughly identifies the nodule but fails to delineate its complete boundary due to posterior acoustic shadowing. The artifact masks the true edge of the nodule, resulting in a segmented region that deviates noticeably from the ground truth. This type of under segmentation is typical when artifacts corrupt the boundary information. These failures are inherently linked to the characteristics of ultrasound imaging: speckle noise, low contrast, and susceptibility to artifacts. While RTS Net performs excellently on nodules with clear boundaries and minimal interference, it still faces challenges in complex scenarios involving heterogeneous backgrounds or artifacts. This analysis underscores that the current model serves as a robust assistive tool for thyroid nodule segmentation rather than a fully automated clinical decision system. Future improvements may incorporate explicit anatomical priors (e.g., thyroid location) or artifact suppression modules to enhance robustness in challenging ultrasound environments.

### 4.7 Discussion with foundation model-based methods

Recently, foundation models such as the Segment Anything Model (SAM) (42) and its medical adaptations (43, 44) have demonstrated remarkable zero-shot segmentation capabilities in both natural and medical images. To comprehensively evaluate the performance of our RTS-Net, we conducted additional experiments to compare it with representative SAM-based methods, ultrasound-specific foundation models, and SAM-assisted semi-supervised methods on our clinical dataset. Table 5 summarizes the quantitative results, where a “Notes” column is added to clarify the model characteristics, experimental settings, and limitations for fair comparison.

**Table 5:** Comparison of different methods on the test dataset.

Method	DICE (%)	Params (M)	Notes
MedSAM (43)	0.8537	93.73	Zero-shot, box prompt; general medical adaptation of SAM
SAMed (44)	0.6179	4.07	Fine-tuned with LoRA; lightweight SAM adaptation
SAM-U (36)	–	–	SAM adaptation with multi-box prompts; uncertainty estimation; no public code
SAMUS (45)	0.8671	130.1	SAM-based ultrasound foundation model; auto-prompting framework
USFM (46)	0.8012	115.46	Universal ultrasound foundation model; label-efficient
SemiSAM+ (47)	–	–	3D semi-supervised model; SAM-assisted lightweight model training
RTS-Net	**0.7371**	**32.25**	**Fully supervised; balanced performance and efficiency**

Bold values indicate the results of our proposed method (RTS-Net).

From the results, MedSAM (43) achieves a high DICE score of 0.8537 in a zero-shot manner with box prompts, showcasing the powerful generalization of large-scale foundation models pre-trained on massive natural and medical image data. However, this excellent performance comes at the cost of a significantly larger parameter count (93.73M), which hinders its deployment on resource-constrained clinical devices such as portable ultrasound scanners or edge computing workstations commonly used in point-of-care settings. Additionally, the reliance on box prompts introduces additional human intervention, which may limit its application in fully automated clinical workflows.

In contrast, SAMed (44), which is fine-tuned with LoRA, drastically reduces the number of parameters to 4.07 M, making it more lightweight and suitable for edge deployment. Unfortunately, this efficiency is accompanied by a notable drop in segmentation accuracy (DICE = 0.6179), indicating that the low-rank adaptation may not fully capture the complex anatomical features and inter-patient variability present in our clinical data. This observation suggests that while parameter-efficient fine-tuning can effectively reduce model size, it may sacrifice the fine-grained details required for reliable medical image segmentation.

SAMUS (45), as an ultrasound-specific foundation model with an auto-prompting mechanism, achieves the highest DICE score of 0.8671 among all compared methods. Its excellent performance benefits from the targeted pre-training on ultrasound images and the auto-prompting design that avoids manual intervention, which fully verifies the advantages of ultrasound-specialized foundation models in clinical ultrasound segmentation tasks. However, its parameter count (130.1M) is even larger than that of MedSAM, which further increases the difficulty of its deployment in resource-constrained clinical scenarios, limiting its practical application value.

USFM (46), another universal ultrasound foundation model, achieves a DICE score of 0.8012 with 115.46M parameters. Its performance is slightly lower than SAMUS and MedSAM but higher than our RTS-Net, which is attributed to its pre-training on a large number of multi-organ and multi-task ultrasound data, leading to strong generalization ability. Similar to SAMUS and MedSAM, the large parameter size of USFM (115.46M) is still a major bottleneck for its deployment in edge clinical devices.

Regarding SAM-U (36), a multi-prompt triggered SAM adaptation method that can be categorized as a foundation model-based approach, although it adopts a multi-prompt mechanism to optimize segmentation performance, its official code is not publicly available, which makes it impossible to reproduce its experimental results and conduct further comparative analysis. Therefore, we only list its relevant information for reference, and focus on the comparison with methods that have public and reproducible results.

As for SemiSAM+ (47), which utilizes SAM to assist the training of lightweight models like U-Net, it is a 3D semi-supervised segmentation model. Due to the fundamental difference between 3D model design and our 2D clinical segmentation task, direct quantitative comparison is not fair or feasible. However, we acknowledge the value of its design idea—efficiently utilizing foundation models to assist lightweight model training—which aligns with the principle of exploring efficient use of existing foundation models rather than training traditional models from scratch. We will prioritize this direction in our future work, combining the advantages of foundation models and lightweight models to further improve the performance and efficiency of clinical segmentation.

Our RTS-Net achieves a balance between segmentation performance and computational efficiency. With a DICE score of 0.7371, it substantially outperforms the lightweight SAMed while maintaining a manageable parameter size of 32.25M—markedly fewer parameters than MedSAM, SAMUS, and USFM. This performance profile positions RTS-Net as a practical and effective solution for clinical segmentation tasks where both accuracy and computational feasibility are critical constraints. The fully supervised training paradigm enables our model to learn task-specific features directly from clinical data, obviating the need for manual prompting and thereby facilitating automated clinical diagnosis. That said, SAM and its related adaptations remain convenient and valuable tools for medical image segmentation, and we will continue to explore the integration of SAM-based strategies in future work to further enhance model performance and applicability.

## 5 Conclusion

In this paper, we propose RTS-Net, an attention-guided thyroid segmentation network tailored for thyroid nodule segmentation in ultrasound images. The core contributions of RTS-Net are threefold. First, we introduce a dual-path attention enhancement mechanism that synergistically combines spatial and channel attention to suppress irrelevant background interference and enhance feature representation in nodule regions, significantly improving the model's ability to capture small nodules and blurred boundaries. Second, we construct a cascaded graph convolution decoding architecture that achieves multi-scale feature pyramid fusion. By incorporating graph convolution blocks, the model captures long-range spatial dependencies and refines feature representations, thereby ensuring precise segmentation boundaries and topological integrity.

Extensive experiments on the public TN3K and DDTI datasets demonstrate that RTS-Net outperforms various advanced segmentation methods, including U-Net, DeepLabv3+, and TransUNet, achieving leading performance in key metrics such as IoU, F1-score, and Accuracy in both in-distribution and cross-dataset settings, which fully validates its strong generalization capability. While real-world clinical scenarios present challenges such as complex noise and low contrast, RTS-Net maintains robust performance, further confirming its practical adaptability. Failure case analysis reveals occasional misidentifications of normal tissues or underestimation of nodule boundaries in the presence of artifacts, highlighting the need for further optimization.

Ablation experiments confirm the individual effectiveness of the proposed dual-path attention mechanism and graph convolution module, as well as their synergistic effects, validating the rationality of our architectural design. In the future, we will explore the integration of foundation models such as SAM to enhance the model's zero-shot generalization ability and reduce reliance on large-scale annotated data. By leveraging SAM's strong feature extraction capabilities and prompt-driven segmentation logic, we aim to address the limitations of RTS-Net in handling complex ultrasound scenarios, further improving segmentation accuracy and clinical applicability.

## Data Availability

The original contributions presented in the study are included in the article/Supplementary material, further inquiries can be directed to the corresponding authors.
