# Supplementary figures and images for: RTS-Net: thyroid nodule segmentation network integrating dual-path attention and graph convolution
Source: Front Med (Lausanne). 2026 Apr 13;13:1785796. doi: 10.3389/fmed.2026.1785796 (PMC13111076; doi:10.3389/fmed.2026.1785796)

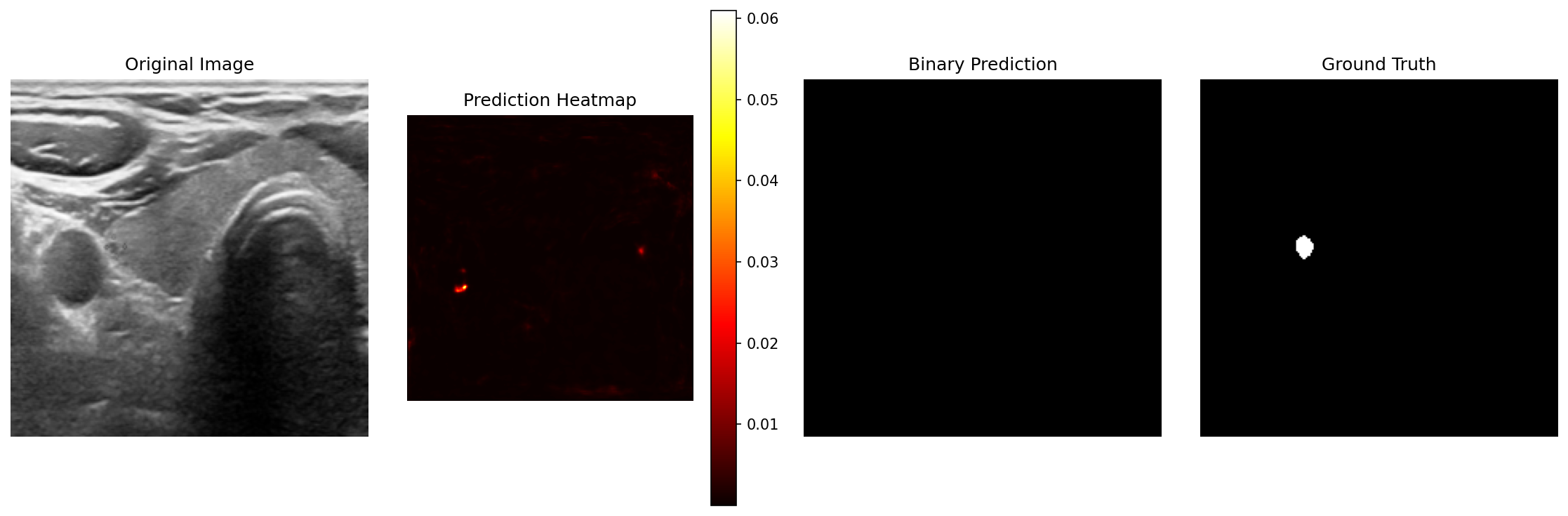

Supplement: Supplementary file 1 [file Data_Sheet_1.zip › 2_RAW_DATA_FOR_RTSNET/Predicted_Masks/clinical dataset/cpfnet/fold0/heatmaps/best_model_['16.png']_heatmap.png]

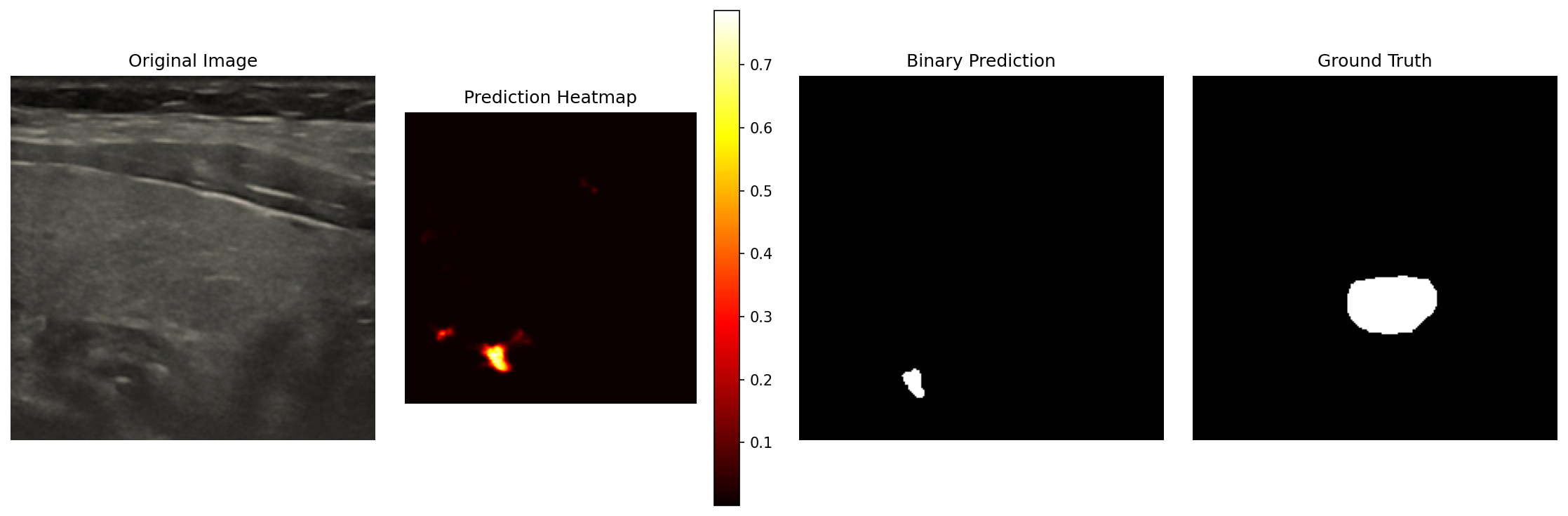

Supplement: Supplementary file 1 [file Data_Sheet_1.zip › 2_RAW_DATA_FOR_RTSNET/Predicted_Masks/clinical dataset/cpfnet/fold0/heatmaps/best_model_['54.png']_heatmap.png]

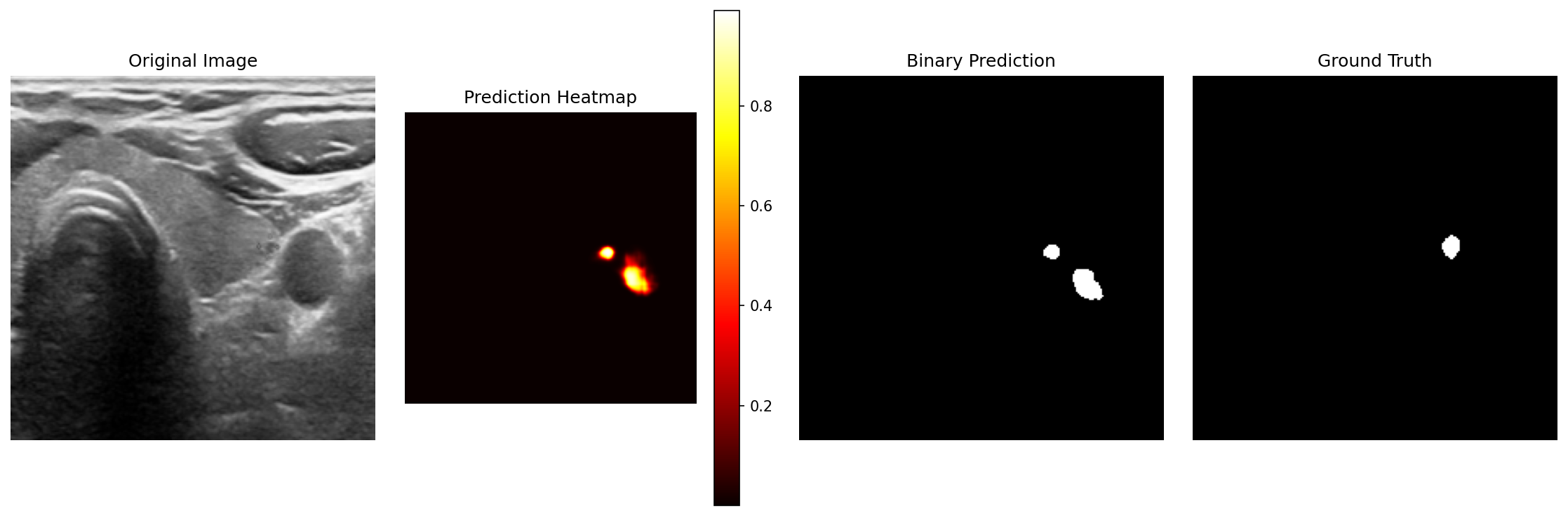

Supplement: Supplementary file 1 [file Data_Sheet_1.zip › 2_RAW_DATA_FOR_RTSNET/Predicted_Masks/clinical dataset/RTSNet/fold0/heatmaps/best_model_['16.png']_heatmap.png]

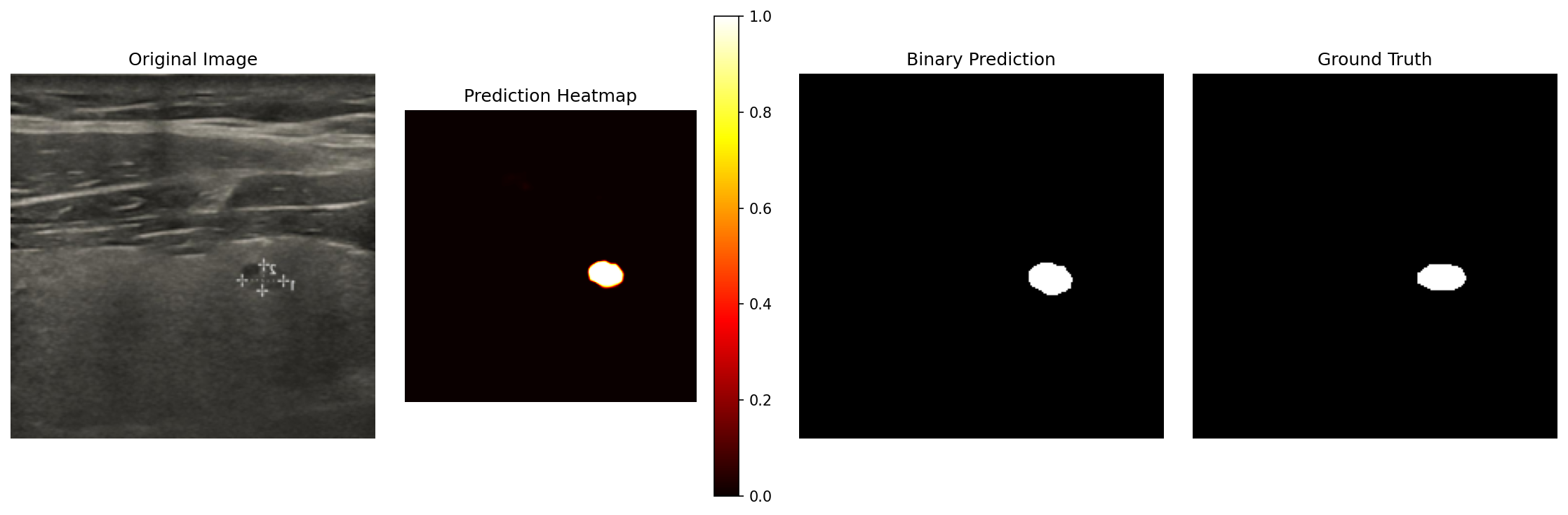

Supplement: Supplementary file 1 [file Data_Sheet_1.zip › 2_RAW_DATA_FOR_RTSNET/Predicted_Masks/clinical dataset/RTSNet/fold0/heatmaps/best_model_['22.png']_heatmap.png]

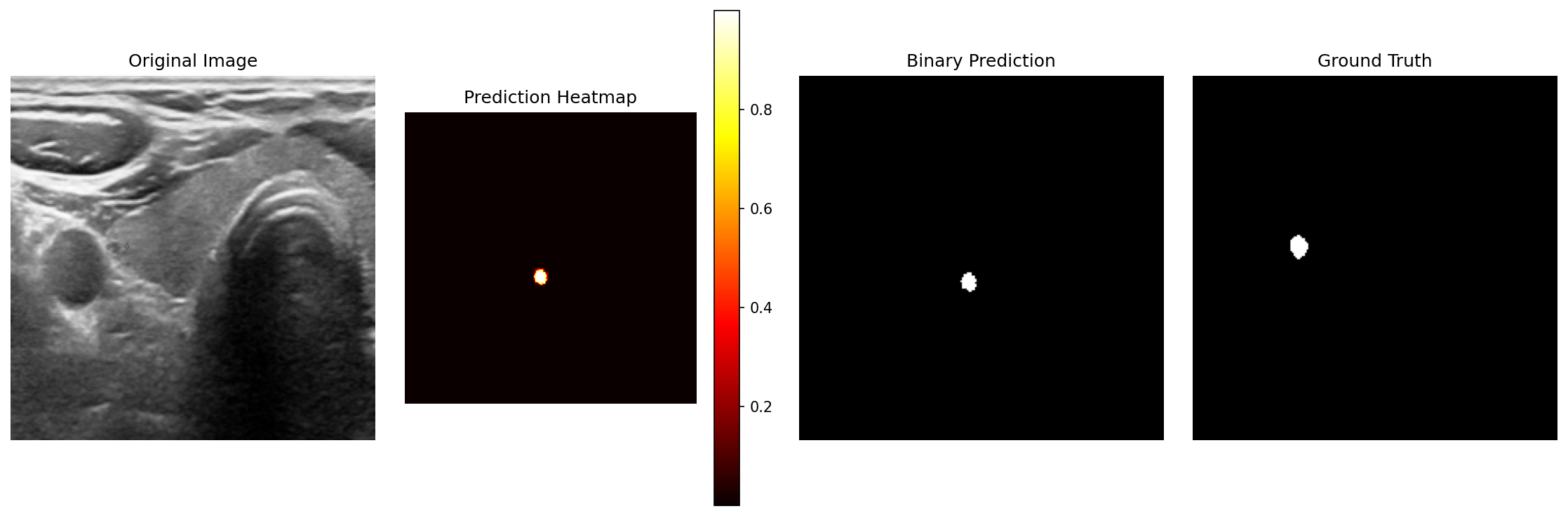

Supplement: Supplementary file 1 [file Data_Sheet_1.zip › 2_RAW_DATA_FOR_RTSNET/Predicted_Masks/clinical dataset/segnet/fold0/heatmaps/best_model_['16.png']_heatmap.png]

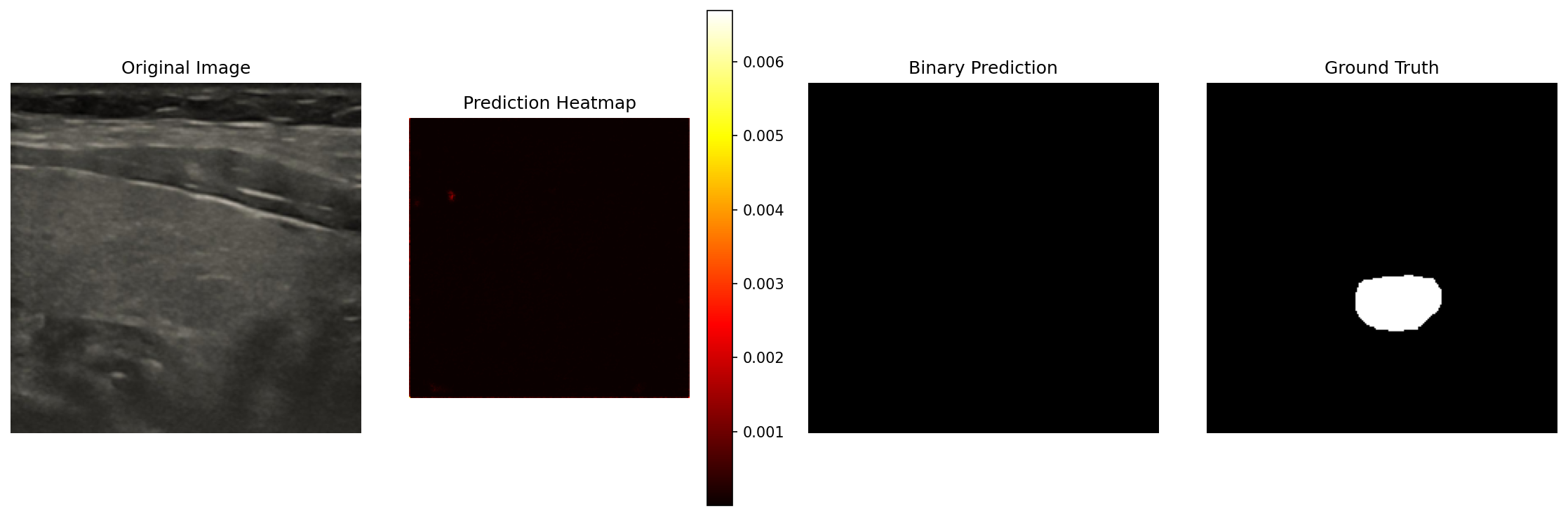

Supplement: Supplementary file 1 [file Data_Sheet_1.zip › 2_RAW_DATA_FOR_RTSNET/Predicted_Masks/clinical dataset/segnet/fold0/heatmaps/best_model_['54.png']_heatmap.png]

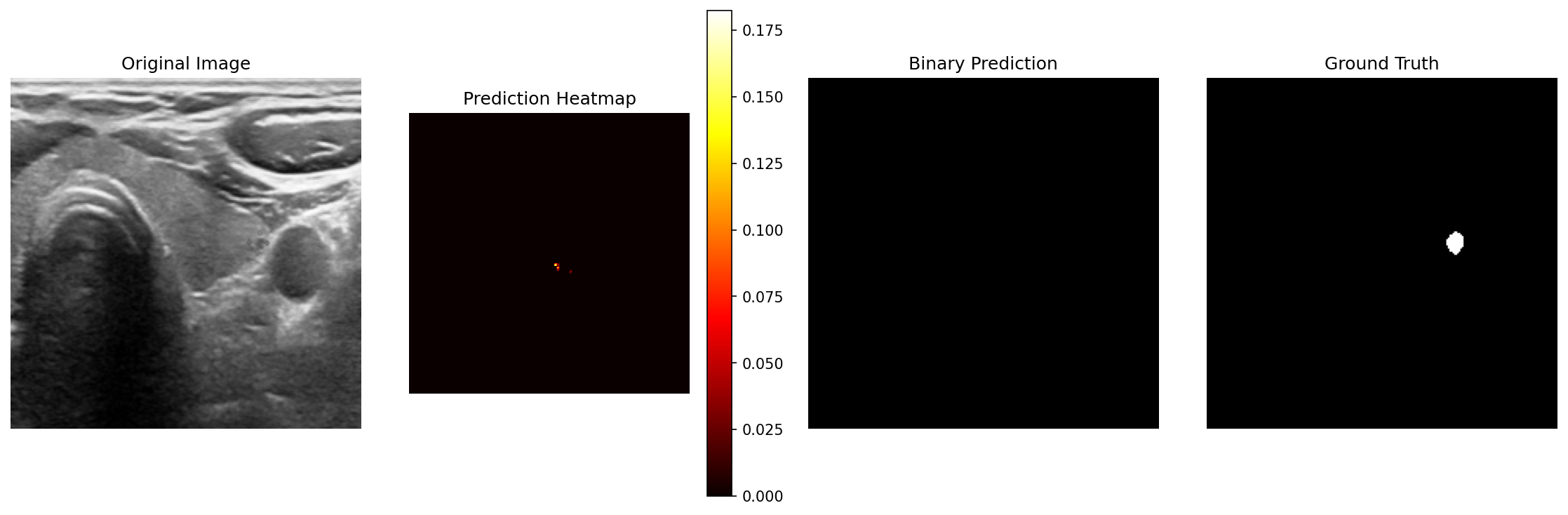

Supplement: Supplementary file 1 [file Data_Sheet_1.zip › 2_RAW_DATA_FOR_RTSNET/Predicted_Masks/clinical dataset/sgunet/fold0/heatmaps/best_model_['16.png']_heatmap.png]

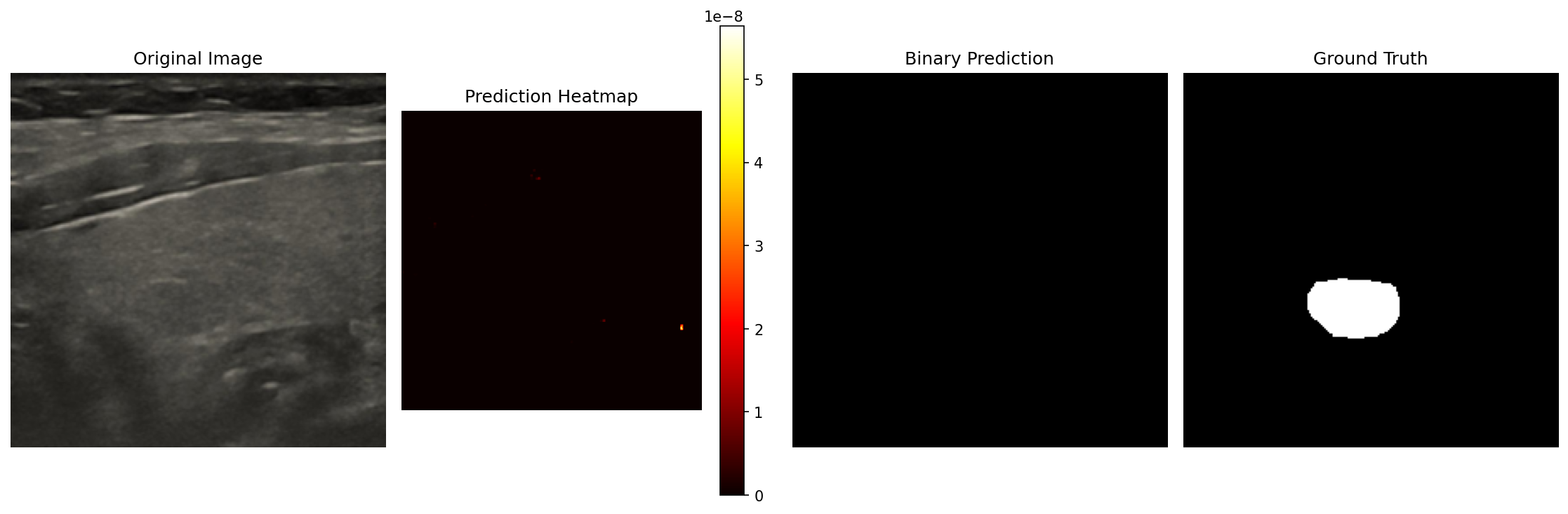

Supplement: Supplementary file 1 [file Data_Sheet_1.zip › 2_RAW_DATA_FOR_RTSNET/Predicted_Masks/clinical dataset/sgunet/fold0/heatmaps/best_model_['54.png']_heatmap.png]

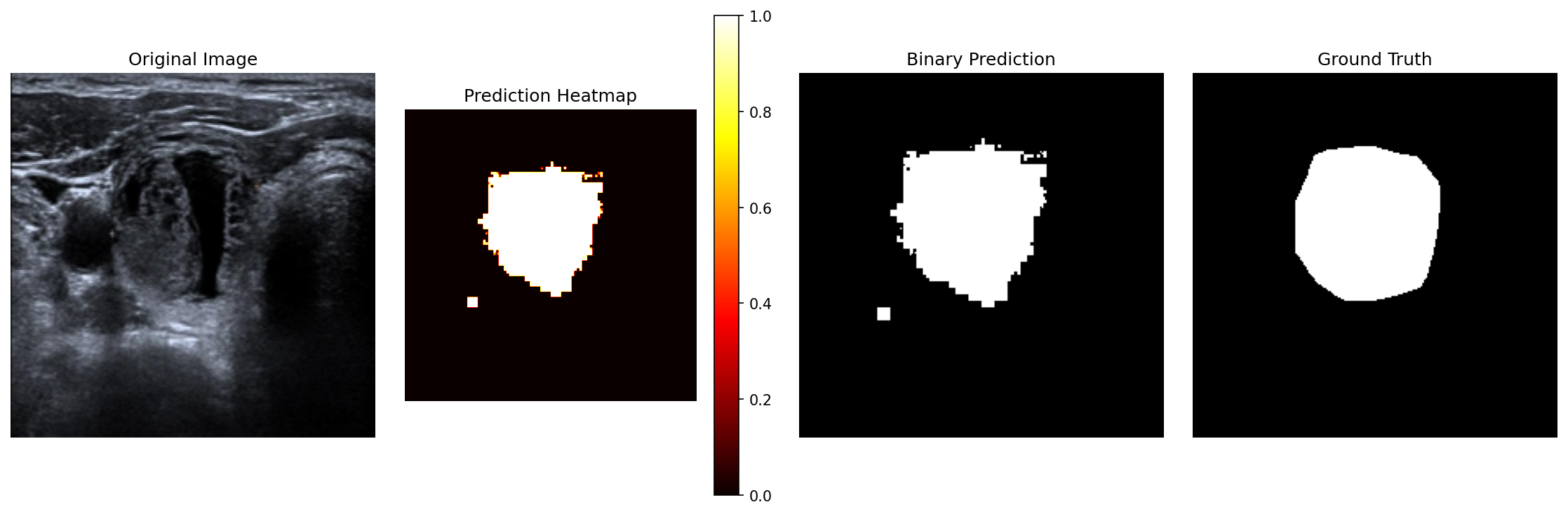

Supplement: Supplementary file 1 [file Data_Sheet_1.zip › 2_RAW_DATA_FOR_RTSNET/Predicted_Masks/clinical dataset/sgunet/fold0/heatmaps/best_model_['60.png']_heatmap.png]

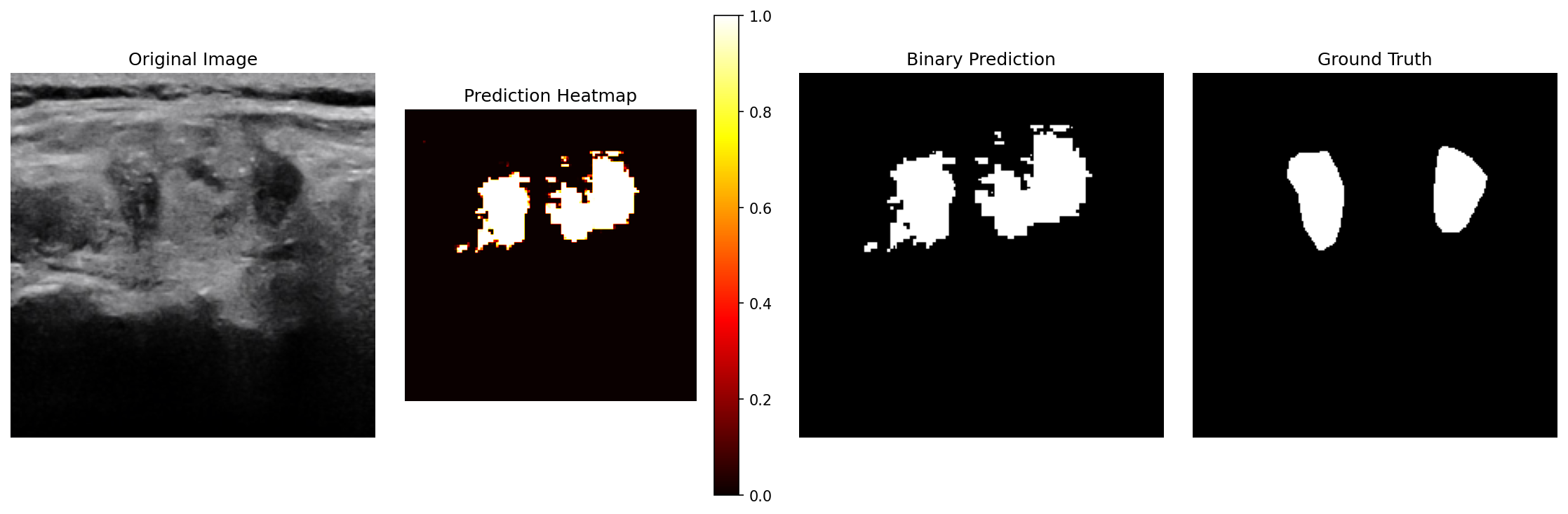

Supplement: Supplementary file 1 [file Data_Sheet_1.zip › 2_RAW_DATA_FOR_RTSNET/Predicted_Masks/clinical dataset/sgunet/fold0/heatmaps/best_model_['68.png']_heatmap.png]

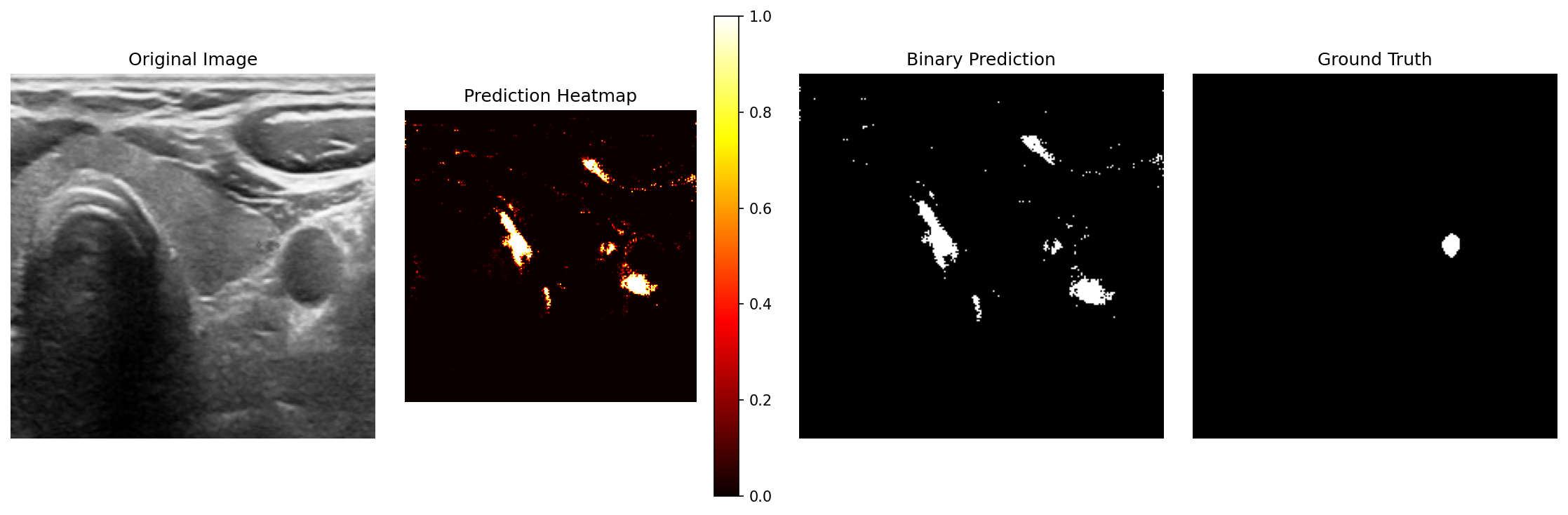

Supplement: Supplementary file 1 [file Data_Sheet_1.zip › 2_RAW_DATA_FOR_RTSNET/Predicted_Masks/clinical dataset/unet/fold0/heatmaps/best_model_['16.png']_heatmap.png]

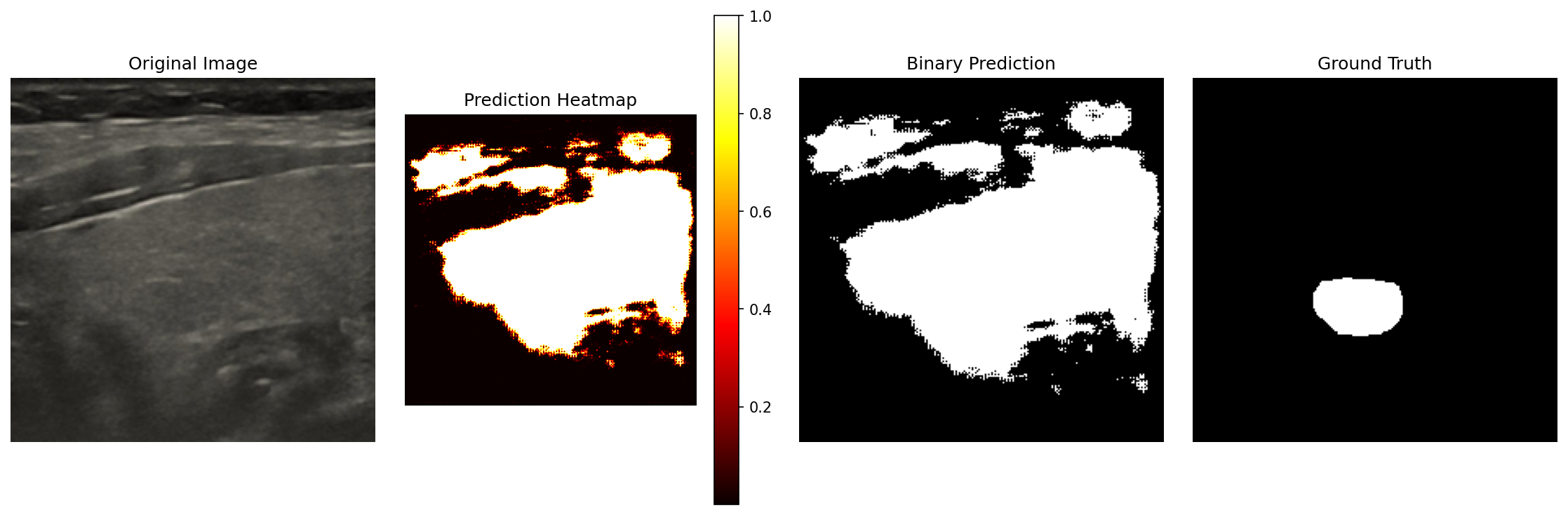

Supplement: Supplementary file 1 [file Data_Sheet_1.zip › 2_RAW_DATA_FOR_RTSNET/Predicted_Masks/clinical dataset/unet/fold0/heatmaps/best_model_['54.png']_heatmap.png]

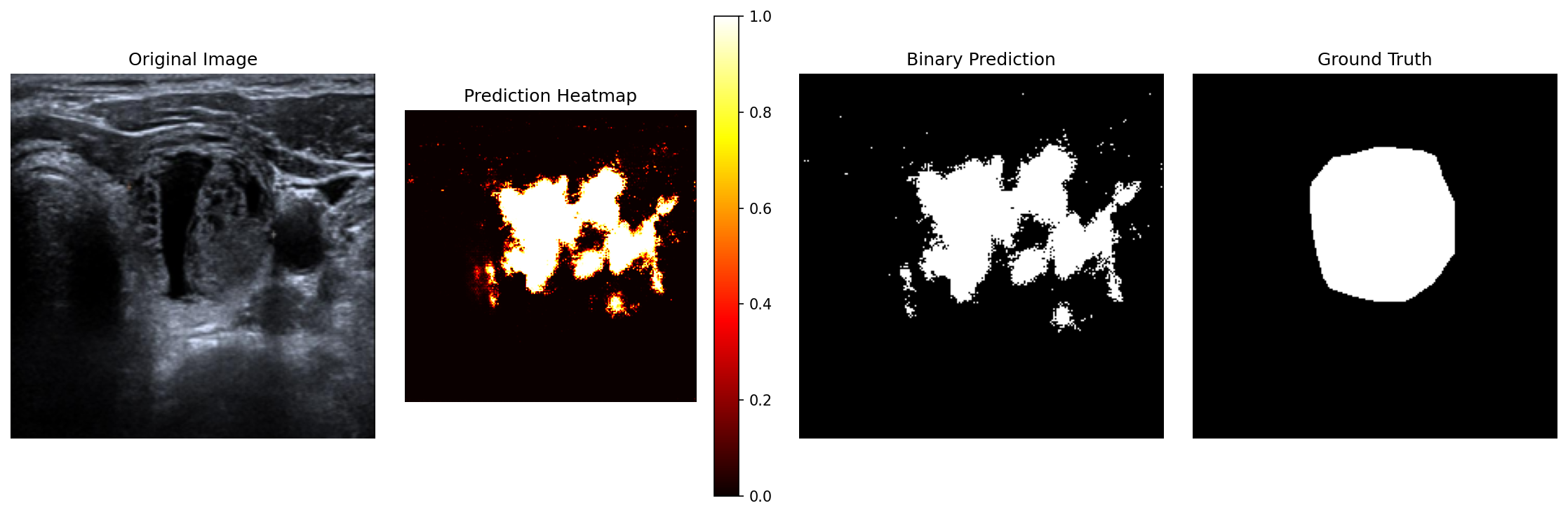

Supplement: Supplementary file 1 [file Data_Sheet_1.zip › 2_RAW_DATA_FOR_RTSNET/Predicted_Masks/clinical dataset/unet/fold0/heatmaps/best_model_['60.png']_heatmap.png]

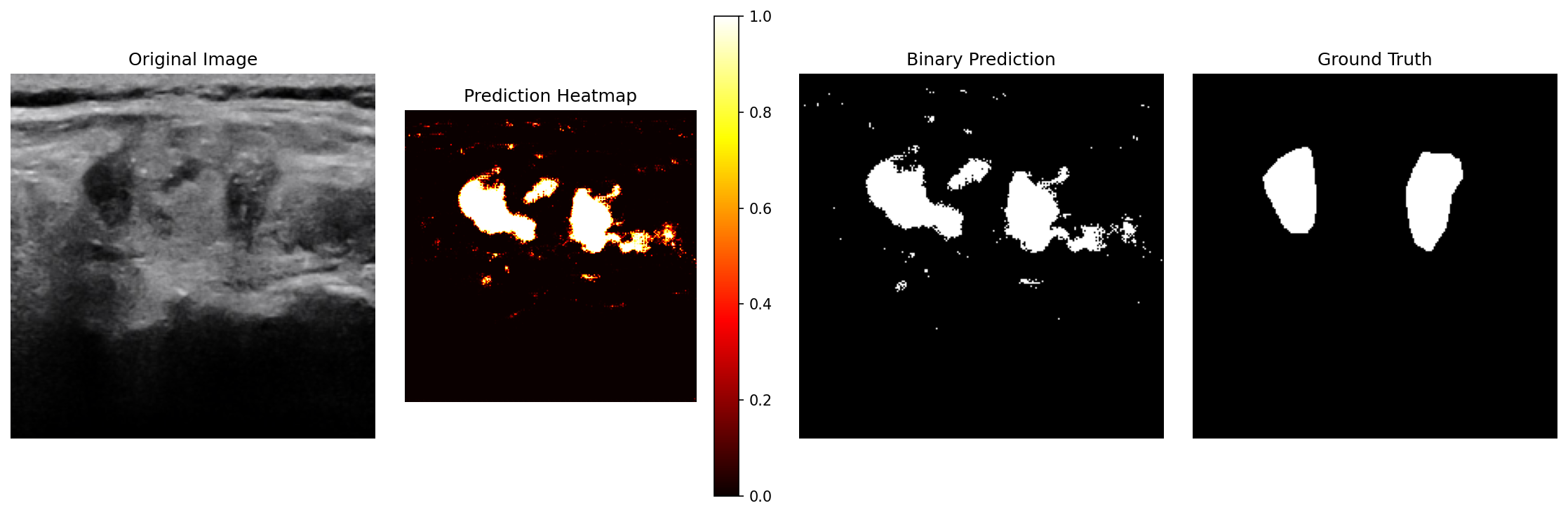

Supplement: Supplementary file 1 [file Data_Sheet_1.zip › 2_RAW_DATA_FOR_RTSNET/Predicted_Masks/clinical dataset/unet/fold0/heatmaps/best_model_['68.png']_heatmap.png]

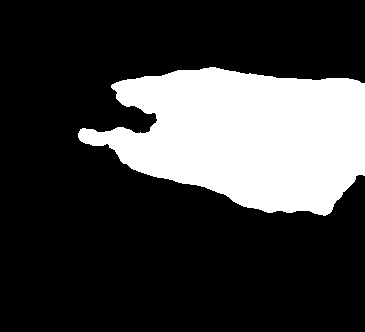

Supplement: Supplementary file 1 [file Data_Sheet_1.zip › 2_RAW_DATA_FOR_RTSNET/Predicted_Masks/TN3K/DeepLabv3+/fold0/0000.jpg]

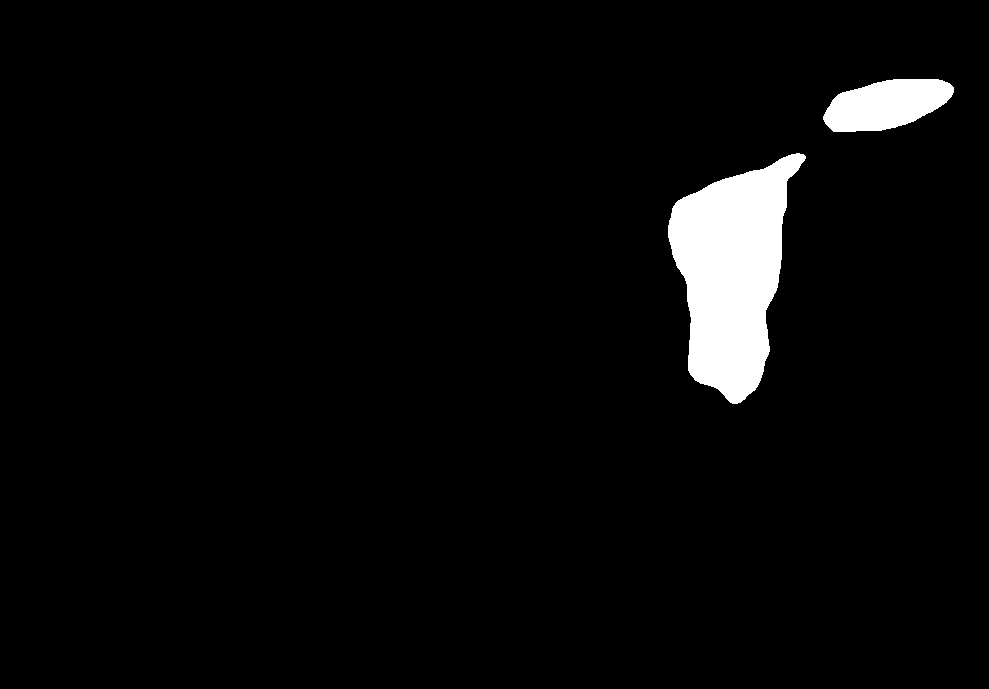

Supplement: Supplementary file 1 [file Data_Sheet_1.zip › 2_RAW_DATA_FOR_RTSNET/Predicted_Masks/TN3K/DeepLabv3+/fold0/0001.jpg]

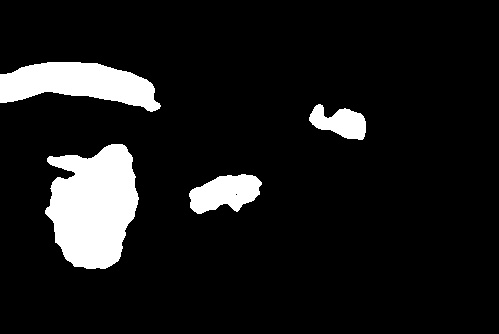

Supplement: Supplementary file 1 [file Data_Sheet_1.zip › 2_RAW_DATA_FOR_RTSNET/Predicted_Masks/TN3K/DeepLabv3+/fold0/0002.jpg]

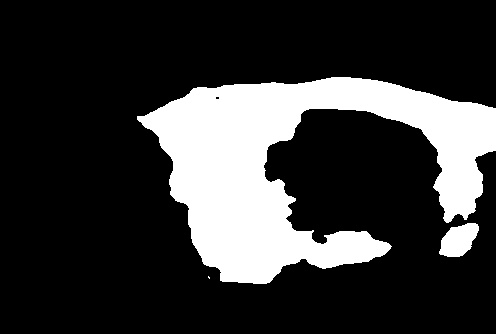

Supplement: Supplementary file 1 [file Data_Sheet_1.zip › 2_RAW_DATA_FOR_RTSNET/Predicted_Masks/TN3K/DeepLabv3+/fold0/0003.jpg]

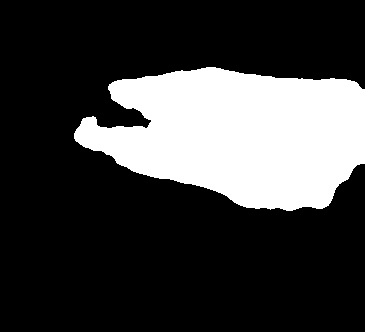

Supplement: Supplementary file 1 [file Data_Sheet_1.zip › 2_RAW_DATA_FOR_RTSNET/Predicted_Masks/TN3K/TransUNet/fold0/0000.jpg]

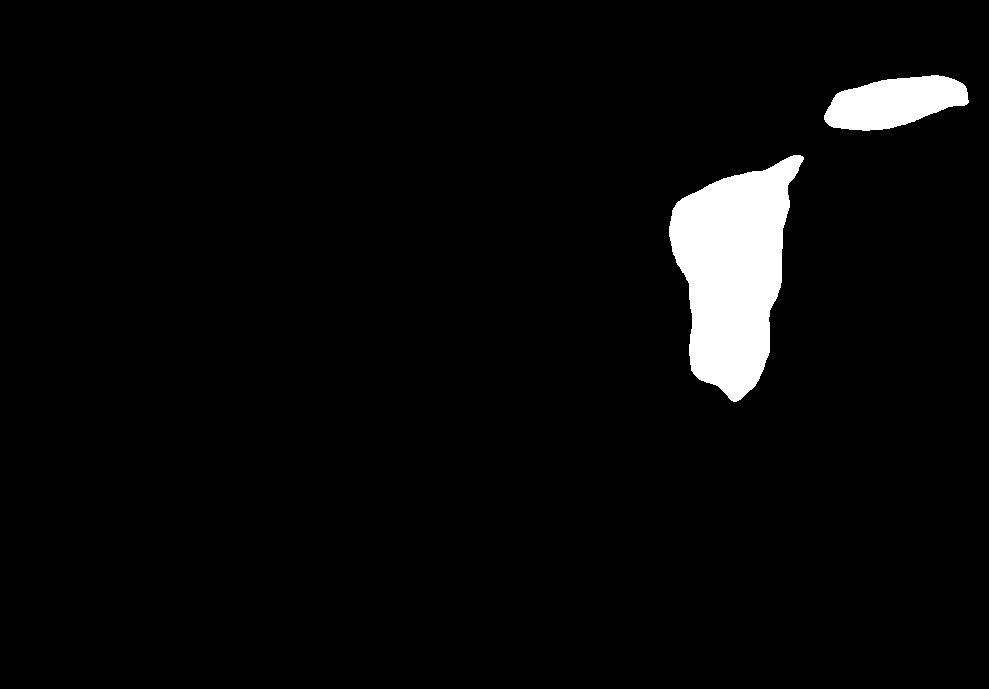

Supplement: Supplementary file 1 [file Data_Sheet_1.zip › 2_RAW_DATA_FOR_RTSNET/Predicted_Masks/TN3K/TransUNet/fold0/0001.jpg]

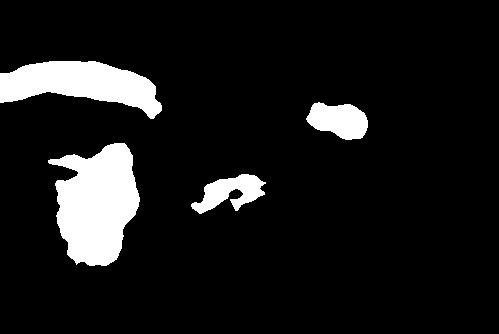

Supplement: Supplementary file 1 [file Data_Sheet_1.zip › 2_RAW_DATA_FOR_RTSNET/Predicted_Masks/TN3K/TransUNet/fold0/0002.jpg]

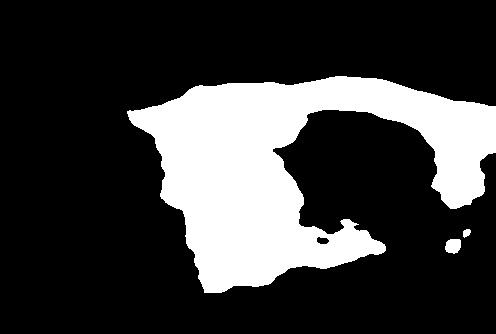

Supplement: Supplementary file 1 [file Data_Sheet_1.zip › 2_RAW_DATA_FOR_RTSNET/Predicted_Masks/TN3K/TransUNet/fold0/0003.jpg]

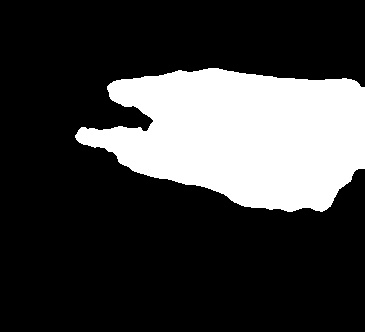

Supplement: Supplementary file 1 [file Data_Sheet_1.zip › 2_RAW_DATA_FOR_RTSNET/Predicted_Masks/TN3K/RTSNet/fold0/0000.jpg]

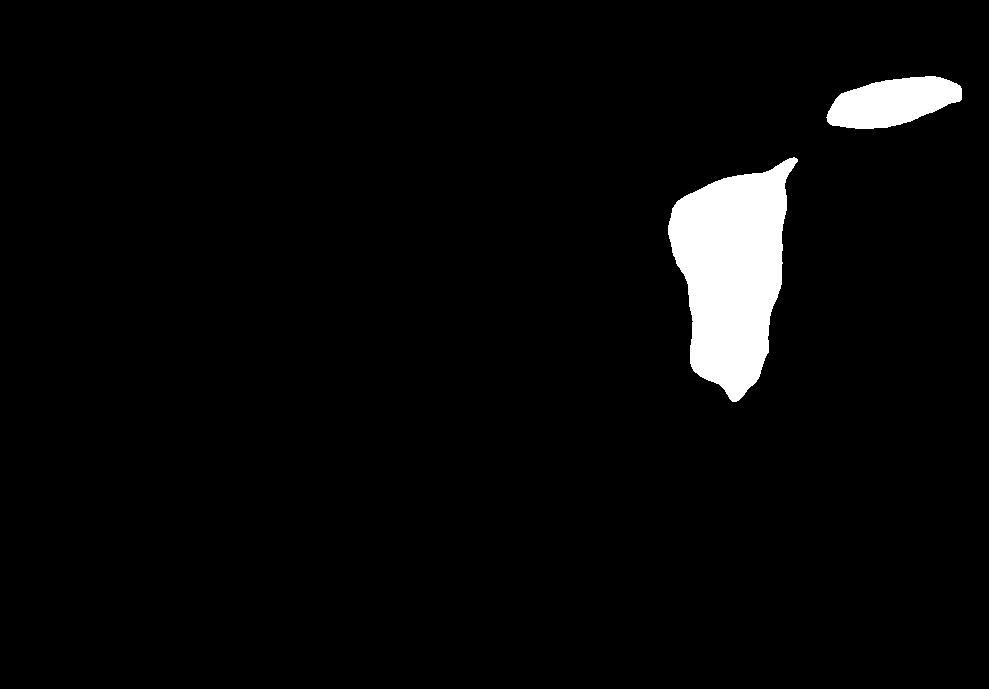

Supplement: Supplementary file 1 [file Data_Sheet_1.zip › 2_RAW_DATA_FOR_RTSNET/Predicted_Masks/TN3K/RTSNet/fold0/0001.jpg]

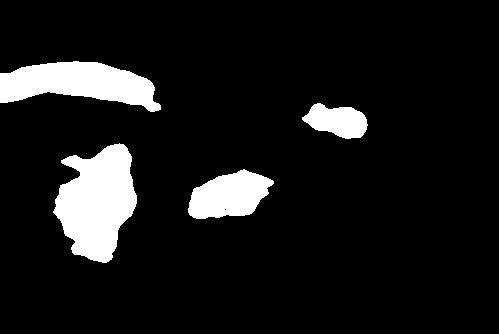

Supplement: Supplementary file 1 [file Data_Sheet_1.zip › 2_RAW_DATA_FOR_RTSNET/Predicted_Masks/TN3K/RTSNet/fold0/0002.jpg]

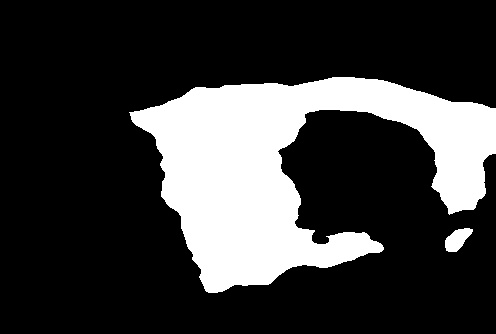

Supplement: Supplementary file 1 [file Data_Sheet_1.zip › 2_RAW_DATA_FOR_RTSNET/Predicted_Masks/TN3K/RTSNet/fold0/0003.jpg]

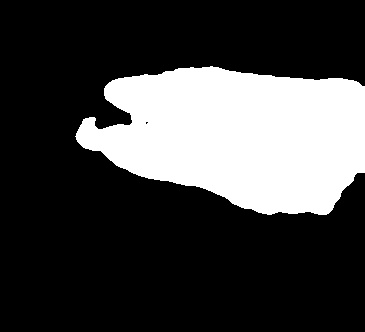

Supplement: Supplementary file 1 [file Data_Sheet_1.zip › 2_RAW_DATA_FOR_RTSNET/Predicted_Masks/TN3K/UNet/fold0/0000.jpg]

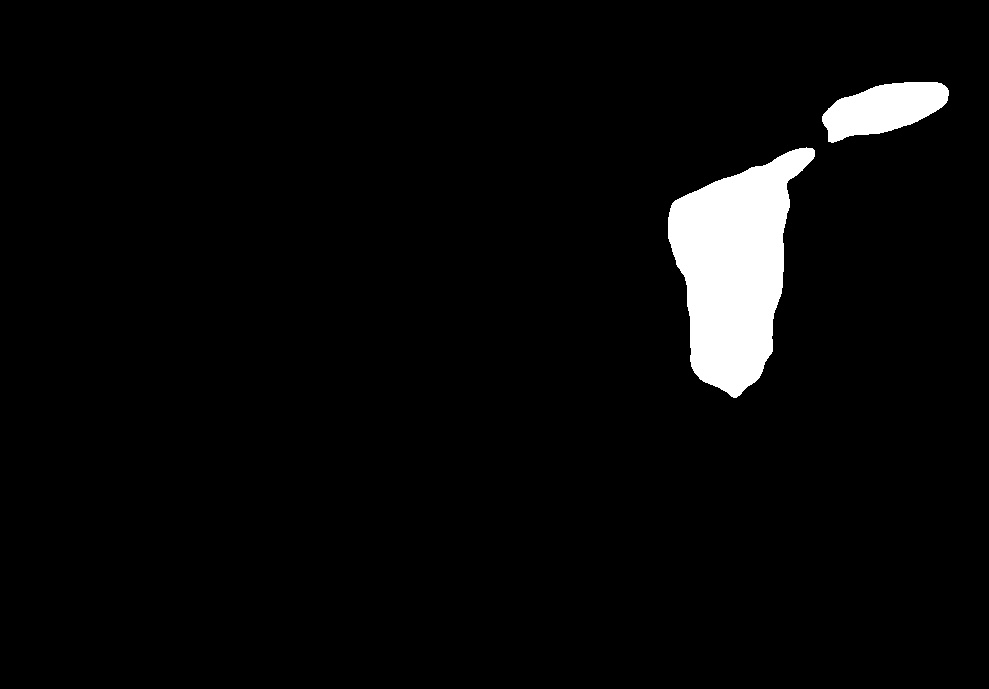

Supplement: Supplementary file 1 [file Data_Sheet_1.zip › 2_RAW_DATA_FOR_RTSNET/Predicted_Masks/TN3K/UNet/fold0/0001.jpg]

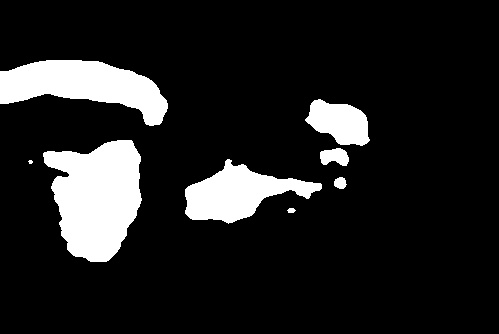

Supplement: Supplementary file 1 [file Data_Sheet_1.zip › 2_RAW_DATA_FOR_RTSNET/Predicted_Masks/TN3K/UNet/fold0/0002.jpg]

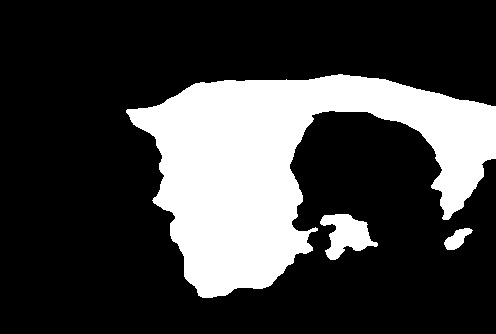

Supplement: Supplementary file 1 [file Data_Sheet_1.zip › 2_RAW_DATA_FOR_RTSNET/Predicted_Masks/TN3K/UNet/fold0/0003.jpg]
